# Landscape Genomic Conservation Assessment of a Narrow-Endemic and a Widespread Morning Glory From Amazonian Savannas

**DOI:** 10.3389/fpls.2018.00532

**Published:** 2018-05-07

**Authors:** Éder C. Lanes, Nathaniel S. Pope, Ronnie Alves, Nelson M. Carvalho Filho, Tereza C. Giannini, Ana M. Giulietti, Vera L. Imperatriz-Fonseca, Waléria Monteiro, Guilherme Oliveira, Amanda R. Silva, José O. Siqueira, Pedro W. Souza-Filho, Santelmo Vasconcelos, Rodolfo Jaffé

**Affiliations:** ^1^Instituto Tecnológico Vale, Belém, Brazil; ^2^Biological Laboratories, Department of Integrative Biology, University of Texas, Austin, TX, United States; ^3^Ciências Biológicas-Botânica Tropical, Universidade Federal Rural da Amazônia/Museu Paraense Emílio Goeldi, Belém, Brazil; ^4^Instituto de Geociências, Universidade Federal do Pará, Belém, Brazil; ^5^Departamento de Ecologia, Universidade de São Paulo, São Paulo, Brazil; ^6^Instituto de Ciências Biológicas, Universidade Federal do Pará, Belém, Brazil

**Keywords:** biodiversity conservation, environmental association test, *Ipomoea*, isolation by resistance, IUCN red list, landscape genetics, RAD-sequencing, SNP

## Abstract

Although genetic diversity ultimately determines the ability of organisms to adapt to environmental changes, conservation assessments like the widely used International Union for Conservation of Nature (IUCN) Red List Criteria do not explicitly consider genetic information. Including a genetic dimension into the IUCN Red List Criteria would greatly enhance conservation efforts, because the demographic parameters traditionally considered are poor predictors of the evolutionary resilience of natural populations to global change. Here we perform the first genomic assessment of genetic diversity, gene flow, and patterns of local adaptation in tropical plant species belonging to different IUCN Red List Categories. Employing RAD-sequencing we identified tens of thousands of single-nucleotide polymorphisms in an endangered narrow-endemic and a least concern widespread morning glory (Convolvulaceae) from Amazonian savannas, a highly threatened and under-protected tropical ecosystem. Our results reveal greater genetic diversity and less spatial genetic structure in the endangered species. Whereas terrain roughness affected gene flow in both species, forested and mining areas were found to hinder gene flow in the endangered plant. Finally we implemented environmental association tests and genome scans for selection, and identified a higher proportion of candidate adaptive loci in the widespread species. These mainly contained genes related to pathogen resistance and physiological adaptations to life in nutrient-limited environments. Our study emphasizes that IUCN Red List Criteria do not always prioritize species with low genetic diversity or whose genetic variation is being affected by habitat loss and fragmentation, and calls for the inclusion of genetic information into conservation assessments. More generally, our study exemplifies how landscape genomic tools can be employed to assess the status, threats and adaptive responses of imperiled biodiversity.

## Introduction

Species conservation assessments have largely neglected measures of genetic diversity, even though genetic diversity ultimately determines the ability to adapt to environmental changes and therefore underpins species long-term persistence (Reed and Frankham, 2003; Allendorf et al., 2012; Jamieson and Allendorf, 2012). For instance, the Red List criteria from the International Union for Conservation of Nature (IUCN), a widely used system to determine conservation priorities across the globe (Rodrigues et al., 2006; IUCN, 2017), does not explicitly consider genetic diversity, emphasizing instead population size, population declines and geographic range (Mace et al., 2008; IUCN, 2012). While the correlation between occurrence range and genetic diversity has been well documented in plants (Hamrick et al., 1991; Gitzendanner and Soltis, 2000; Cole, 2003; Leimu et al., 2006; Gibson et al., 2008), census size and population declines are not consistent predictors of genetic diversity. For instance, reductions in population size or geographic range may not result in diminished genetic diversity (Frankham, 1995; Honnay and Jacquemyn, 2007; Aguilar et al., 2008; Vranckx et al., 2012).

Two recent studies evaluated genetic diversity patterns across IUCN Red List Categories. Rivers et al. (2014) calculated the loss of genetic diversity under simulated range loss for two plant species in Madagascar. They found a strong relationship between loss of genetic diversity (AFLP allelic richness) and range, and a correspondence between levels of genetic diversity and thresholds for “non-threatened” vs. “threatened” IUCN Red List Categories. However, genetic diversity distinction between threatened categories was less evident, as the amount of genetic diversity at a specific threat category was not consistent across criteria (Rivers et al., 2014). Similarly, Willoughby et al. (2015) gathered 1941 microsatellite datasets from wild populations of five vertebrate classes. They then compared empirical estimates of heterozygosity and allelic richness between threatened and non-threatened species, and found that genetic diversity is generally reduced in threatened species. However, when they considered which of the IUCN Red List Criteria (including declining population size, species range extent, and the number of mature individuals) are most effective at identifying genetically depauperate species, they found that the existing criteria failed to systematically identify populations with low genetic diversity (Willoughby et al., 2015). Thus, threatened species generally show reduced genetic diversity compared to non-threatened ones (Spielman et al., 2004), but the IUCN Red List Criteria approach does not systematically prioritize the conservation of species with low genetic diversity (Rivers et al., 2014; Willoughby et al., 2015). Additionally, the genetic consequences of habitat degradation and fragmentation in relation to the IUCN Red List Categories remain largely unexplored.

As DNA sequencing and bioinformatic processing have become more affordable, genetic data have rapidly accumulated across a wide range of taxa (Vranckx et al., 2012; Frankham, 2015; Garner et al., 2017), many of which have never been assessed using the IUCN Red List Criteria. It is thus essential to integrate such data with the IUCN Red List Criteria in order to gain deeper insights into the susceptibility and survival perspectives of imperiled biodiversity and make optimal conservation decisions (Laikre, 2010; Shafer et al., 2015; Pierson et al., 2016). However, no conservation assessment has yet compared genetic diversity and gene flow patterns between species from different IUCN Red List Categories (Rivers et al., 2014; Willoughby et al., 2015). Moreover, genomic tools have never been employed to validate this categorization.

Here we address these important knowledge gaps by performing the first genomic assessment, to our knowledge, of genetic diversity, gene flow, and patterns of local adaptation in tropical plants belonging to different IUCN Red List Categories. We chose two congeneric and sympatric morning glories (Convolvulaceae) from Amazonian savannas, one of the least known, highly threatened and under-protected tropical ecosystems (de Carvalho and Mustin, 2017). Tropical grassland mosaics associated with rocky outcrops are known to be worldwide hotspots of endemism, and the ancient inselbergs immersed in the Amazon forest are among the most iconic representatives of this ecoregion in South America. The banded iron formations known as Cangas, notorious for their extremely nutrient-poor, metal rich, flooding-prone soils, have also attracted substantial attention from mining companies given that they harbor one of the world's largest deposits of high-grade iron ore (Skirycz et al., 2014; Silveira et al., 2015; Viana et al., 2016).

Our model species were *Ipomoea cavalcantei*, a narrowly-distributed plant classified as *endangered*, and *I. maurandioides*, a species of *least concern* extending from Argentina, Paraguay and Bolivia throughout Brazil (Martinelli and Moraes, 2013; Simão-Bianchini et al., 2016; Wood and Scotland, 2017) (until recently this species was considered *I. carajasensis*, see Data Sheet 1). Although the pollination biology of our study species is yet to be systematically assessed, floral morphology (Simão-Bianchini et al., 2016) suggests that the small lilac flowers of *I. maurandioides* are pollinated by insects while hummingbirds pollinate the red flowers with exerted stamens of *I. cavalcantei* (Galetto and Bernardello, 2004). Fruit morphology, on the other hand, suggests wind-mediated seed dispersal, as in another congeneric species occurring in a neighboring region (Griz and Machado, 2001; Simão-Bianchini et al., 2016). The narrow-endemic *I. cavalcantei* is a flagship species for tropical conservation since its unique habitat is jeopardized by some of the world's biggest mining projects (Souza-Filho et al., 2016; Viana et al., 2016), which involve deforestation of montane savannas, soil removal, and other types of environmental disturbances. The close phylogenetic proximity and contrasting distribution ranges of these two species offer an ideal opportunity to assess genetic diversity and the consequences of habitat loss and fragmentation on plants sharing a similar evolutionary history but belonging to different IUCN Red List Categories. Moreover, the use of thousands of genetic markers broadly distributed across the entire genome of our study species, provides high resolution measures of genetic diversity and robust estimates of gene flow and signatures of adaptation.

We hypothesized that extent of occurrence (IUCN Red List Criterion B, see Data Sheet 1) would determine genetic diversity, gene flow, and susceptibility to habitat loss and fragmentation, thereby influencing patterns of local adaptation. We thus formulated the following predictions: (i) With a narrow distribution range *I. cavalcantei* is likely to show lower genetic diversity and higher spatial genetic structure across different montane savanna highlands than the analyzed populations of the broadly distributed *I. maurandioides*; (ii) Gene flow is probably affected by the structure of natural landscapes in both species, but landscape modifications by mining are more likely to hinder gene flow in the narrowly-distributed *I. cavalcantei*. Additionally, landscape effects on gene flow are expected to show time-lags determined by the generation time of each species (~20 years for *I. cavalcantei* and ~3 years for *I. maurandioides*); (iii) Although similar adaptations to the extreme montane savanna environments are expected in both species, genetic drift is likely stronger in the narrowly-distributed species, thus slowing adaptive responses. We therefore expect to find weaker adaptive signals (a lower proportion of candidate loci) in *I. cavalcantei*.

## Materials and methods

### Sampling, DNA extraction and genome size estimation

**Figure 1:**
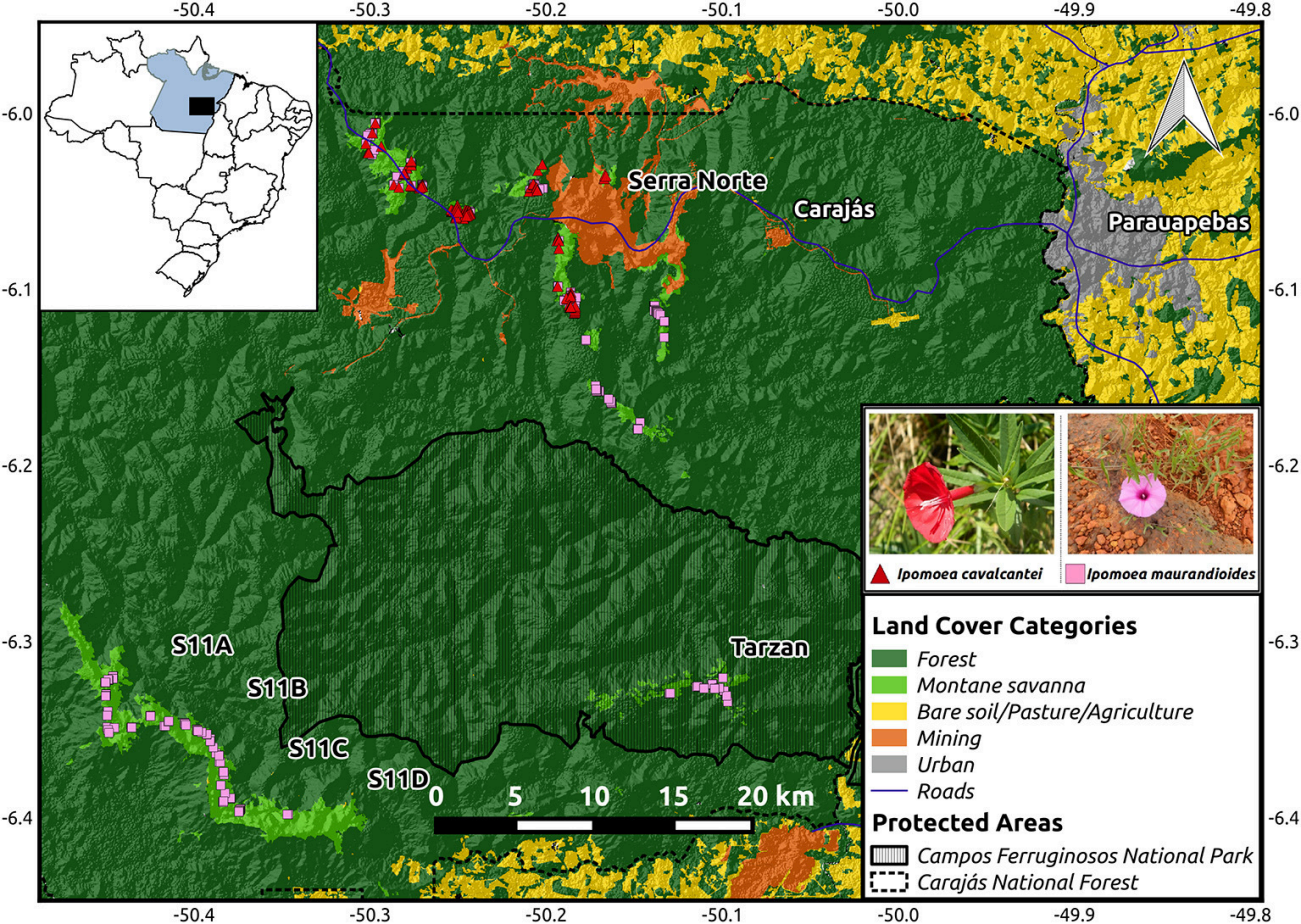
Map of the study region showing the location of the collected samples from *I. cavalcantei* and *I. maurandioides*. The location of the Carajás Mineral Province within Brazil is shown on the upper left corner. An elevation map (from USGS Earth Explorer) is shown overlaid with a land cover color map (from Souza-Filho et al., 2016), and roads (from IBGE). Coordinates are shown in decimal degrees.

We sampled individual plants distributed across our study region, which comprised the full natural occurrence range of *I. cavalvantei*, and all major montane savanna highlands of the Carajás mineral province where *I. maurandioides* is found (Simão-Bianchini et al., 2016; Figure 1). We note that our study region is located at the northern edge of *I. maurandioides* large distribution range (see Data Sheet 1). Leaf tissue samples of 122 individuals of *I. cavalcantei* and 254 individuals of *I. maurandioides* were collected along trails in 2016 (SISBIO collection permit N. 48272-4), observing a minimum distance of 10 m between samples to avoid re-sampling the same individuals (sample coordinates are provided in Data Sheet 2). Samples were preserved in 10 mL of a NaCl-saturated solution of 2% CTAB (Rogstad, 1992), and later frozen at −80°C. Total DNA was extracted using the DNeasy Plant Mini Kit (Qiagen, EUA), DNA concentration quantified using the Qubit High SensitivityAssay kit (Invitrogen), and DNA integrity assessed through 1.2% agarose gel electrophoresis. All DNA samples were adjusted to a final concentration of 5 ng/μL in a final volume of 30 μL. We used flow cytometry to estimate genome size in *I. maurandioides*, as it was already available for *I. cavalcantei* (Babiychuk et al., 2017). Nuclei were obtained from fresh leaf tissues chopped in general purpose buffer with 1% Triton X-100 and 1% PVP-30 (Loureiro et al., 2007). All the steps were conducted on ice until events acquisition on a PI fluorescence mean under a 585/42 bandpass filter. Triplicates of 1000 PI stained nuclei were finally analyzed on a BD FACS Aria II flow cytometer, using a 488 nm laser and *Petroselinum crispum* as an internal reference standard (2 C = 4.50 pg) (Obermayer et al., 2002).

### RAD sequencing and SNP discovery

DNA samples were shipped to SNPSaurus (http://snpsaurus.com/) for sequencing and bioinformatic analyses. Briefly, nextRAD genotyping-by-sequencing libraries were prepared (Russello et al., 2015), considering genome sizes (both species showed a similar genome size, Table S1 and Figure S1). Genomic DNA was first fragmented with Nextera reagent (Illumina, Inc), which also ligates short adapter sequences to the ends of the fragments. The Nextera reaction was scaled for fragmenting 10 ng of genomic DNA, although 15 ng of genomic DNA was used for input to compensate for the amount of degraded DNA in the samples and to increase fragment sizes. Fragmented DNA was then amplified, with one of the primers matching the adapter and extending 9 nucleotides into the genomic DNA with the selective sequence GTGTAGAGC (only fragments starting with a sequence that could be hybridized by the selective sequence of the primer were efficiently amplified). The nextRAD libraries were then sequenced on an Illumina HiSeq 4000 (University of Oregon). Each species was sequenced on three lanes with 150 bp reads.

Reads were trimmed using *BBMap tools* (http://sourceforge.net/projects/bbmap/; ash bbmap/bbduk.sh in = $file out = $outfile ktrim = r k = 17 hdist = 1 mink = 8 ref = bbmap/resources/nextera.fa.gz minlen = 100 ow = t qtrim = r trimq = 10) and a *de novo* reference was created by collecting 10 million reads evenly from the samples, excluding reads that had counts fewer than 10 or more than 400. The remaining loci were then aligned to each other to identify alleles. All reads were mapped to the reference with an alignment identity threshold of 95% using *BBMap tools* (bbmap). Genotype calling was done using *Samtools* and *bcftools* (https://github.com/samtools/samtools; samtools mpileup -gu -Q 10 -t DP,DPR -f ref.fasta -b samples.txt | bcftools call -cv - >genotypes.vcf), and the resulting set of genotypes was filtered to remove loci with a minor allele frequency of <2.5%. Loci with more than 30% missing genotypes, or that were heterozygous in all samples or contained more than 2 alleles in a sample (suggesting collapsed paralogs) were removed. The absence of artifacts was checked by counting SNPs at each read nucleotide position and determining that SNP number did not increase with reduced base quality at the end of the read. This pipeline was carried out separately for each species, with a *de novo* reference created separately for each (genotype and sequence data are available in FigShare: https://doi.org/10.6084/m9.figshare.6100004.v1).

### Genetic diversity and population structure

To address our first prediction we estimated genetic diversity and assessed spatial genetic structure. The R package r2vcftools (https://github.com/nspope/r2vcftools) - a wrapper for VCFtools (Danecek et al., 2011) - was used to perform final quality control on the genotype data (R version 3.4.3). We filtered loci for quality (Phred score >50), read depth (20 - 200), linkage disequilibrium (LD, *r*^2^ < 0.5), and strong deviations from the Hardy Weinberg Equilibrium (HWE, *p* < 0.0001). Additionally, we removed any potential loci under selection detected through environmental association and *F*_*ST*_ outlier tests (see details below). The resulting sets of neutral and independent loci were then used to calculate genetic relatedness (Yang et al., 2010), expected heterozygosity (*H*_*E*_), inbreeding coefficients (*F*), mean per-site nucleotide diversity (π) and Tajima's D (*D*). Effective population size (*N*_*e*_) was also estimated employing the linkage disequilibrium approach of NeEstimator 2.0.1 and a threshold lowest allele frequency value of 0.05 (Do et al., 2014). Two complementary genetic clustering software were used to assess population structure: The *snmf* function of the LEA (v2.0) package (Frichot et al., 2014; Frichot and François, 2015) and Admixture (Alexander et al., 2009). The number ancestral populations (*k*) was allowed to vary between 1 and 10, with 10 replicate runs for each *k*-value, and the best *k* was chosen based on cross-entropy and cross-validation errors (Frichot et al., 2014). Population structure of *I. maurandioides* was assessed a second time, considering only the samples originating from Serra Norte (where the species is sympatric with *I. cavalcantei*).

### Landscape genetic analyses

To tackle our second prediction we first tested for historical effects and then assessed fine-scale spatial genetic structure and isolation by landscape resistance (IBR) (Balkenhol et al., 2016). We tested for historical effects of size and isolation of the montane savanna highlands were our species occur, estimating area (m^2^) and isolation (distance to the nearest highland in meters) using a pre-mining (1984) mapping of montane savanna highlands (Souza-Filho et al., 2016). We then ran mixed effects linear models containing individual-level genetic diversity estimates (*H*_*E*_ and *F*) as response variables, area and isolation as predictors, and highland ID as a random effect (see Figure S2). Likelihood ratio tests (α = 0.05) were then employed to compare reduced models without each predictor variable, with full models containing them. All models were validated by plotting residual vs. fitted values and by checking for multicollinearity and residual autocorrelation.

We assessed fine-scale spatial genetic structure by quantifying spatial autocorrelation in genetic relatedness. To do so we used local polynomial fitting (LOESS) of pairwise relatedness to pairwise geographic distance (https://github.com/rojaff/Lplot; Bruno et al., 2008). We then evaluated the contribution of land cover, elevation, terrain roughness, temperature, precipitation and geographic distance in explaining patterns of gene flow (Balkenhol et al., 2016). Using detailed land cover classification maps (Souza-Filho et al., 2016) we built six resistance surfaces, where we attributed different resistance values to each one of the main land cover classes (montane savanna, forest and mine). Each resistance surface thus represents a quantitative hypothesis about landscape resistance to gene flow. We first attributed low resistance values (0.1) to each main land cover class and high resistance values (0.9) to all other classes. Subsequently we did the opposite, attributing high resistance values to each main land cover class and low resistance values to all other classes. By so doing, each main land cover class was modeled as a high and a low permeability matrix. To account for possible time-lag effects (Landguth et al., 2010) we constructed similar resistance surfaces using land cover maps from three different decades (1994, 2004, and 2013, Figure S2). We chose this time span considering observations made by botanists from the Carajás Zoo Botanical Park, whom have planted both species for public exhibitions and estimate generation times of ~20 years for *I. cavalcantei* and ~3 years for *I. maurandioides*. Since elevation, terrain roughness, mean temperature and mean precipitation have been found to influence gene flow in other plant species (Dyer, 2016), we constructed resistance surfaces employing both the raw and inverted rasters for each one of these variables (see Table S2 for spatial data specifications). Finally, we used a land cover map to create a null model for isolation by resistance (isolation by geographic distance), where all pixels were coded with identical resistance values (0.1). All rasters were set to Universal Transverse Mercator (UTM) projection and cropped to the extent of sample locations plus a buffer area of 5 km to minimize border effects (Jaffé et al., 2015). Using the program Circuitscape V4.0 (McRae, 2006) we then calculated pairwise resistance distances between all samples, employing all the resistance surfaces described above. Due to Circuitscape's computing limitations we replaced pixels containing zero values with 0.001. To assess IBR we fit mixed-effects regression models using penalized least squares and a correlation structure designed to account for the non-independence of pairwise distances (maximum-likelihood population effects or MLPE: https://github.com/nspope/corMLPE; Clarke et al., 2002). Yang's Relatedness between pairs of individuals (Yang et al., 2010) was used as response variable. Similar measures of relatedness have been found to be highly accurate as individual-based genetic distance metrics for landscape genetic studies (Shirk et al., 2017). Predictor variables included the different resistance distances (geographic distance, land cover, elevation, roughness, temperature and precipitation). To reduce the number of predictors we first selected which of the land cover models best explained genetic relatedness, using the sample size corrected Akaike Information Criterion (AICc) to compare univariate models from all years (1994, 2004, and 2013). Similarly, we compared univariate models containing resistance distances from raw and inverted rasters for each one of the remaining predictor variables. We then built full models for each species containing the best land cover surfaces (with lowest AICc) along with the best surfaces from the remaining predictors, and compared all models containing non-collinear predictors (*r* < 0.6) using the *dredge* function from the MuMIn (v1.4) package (https://github.com/rojaff/dredge_mc; Barton, 2015). Models with a ΔAICc < 2 were included in the set of best models, and likelihood ratio tests (LRT) were performed to assess the influence of the inclusion of each predictor variable on the best model's log-likelihood. Finally, model averaging across the set of best models was used to compute parameter estimates that account for uncertainty in model selection (Burnham and Anderson, 2002; Jaffé et al., 2016).

### Identification of putative adaptive loci

Finally, we employed environmental association and *F*_*ST*_ outlier tests to address our third prediction. To identify putative adaptive loci we employed sets of loci filtered only by quality and depth (as described above), but not for LD or HWE. Adaptive signals were evaluated across all SNPs, even though many contigs contained more than one SNP. We thereby avoided the arbitrary exclusion of SNPs and the loss of any potentially adaptive signal. Linkage disequilibrium was nevertheless low within our 150 bp contigs (Figure S3), so most SNPs were independent markers. We used latent factor mixed models (LFMM) to identify possible associations between SNPs and environmental variables, while accounting for the underlying population structure (Frichot et al., 2013; De Kort et al., 2014; Rellstab et al., 2015). We first ran a principal component analysis (PCA) using the 19 bioclimatic variables retrieved from WorldClim plus SRTM Elevation (Table S2), and selected the three variables showing the strongest correlation with the first three PCA axes (which explained 85 and 84% of total variance in *I. cavalcantei* and *I. maurandioides* respectively). These were minimum temperature of coldest month, precipitation of warmest quarter, and precipitation of wettest quarter for *I. cavalcantei;* and minimum temperature of coldest month, precipitation of wettest quarter, and precipitation of coldest quarter for *I. maurandioides*. Correlations between these variables were weak (*r* < 0.3), except for minimum temperature of coldest month and precipitation of wettest quarter in *I. maurandioides* (*r* = 0.67). We then used these variables to run LFMM using the LEA R package. Since incorrect assumptions about underlying demographic structure can increase both Type I and Type II errors (Storfer et al., 2018), we ran LFMM using multiple *k*-values (where *k* was the optimum number of ancestral populations detected), and then intersected candidate loci detected across all runs. Models were ran for *k* ± 2 latent factors (*I. cavalcantei*: *k* = 1, 2, and 3; *I. maurandioides*: *k* = 2, 3, 4, 5, and 6; see results below). We used 10,000 iterations, a burn-in of 5,000 and five runs per environmental variable (Frichot and François, 2015). Finally, *p*-values were adjusted using the genomic inflation factor (λ) and false discovery rates were set using the Benjamini-Hochberg algorithm (Benjamini and Hochberg, 1995). We only considered as candidate loci those shared between all runs (assuming different *k*-values). In addition, we ran genome scans for selection using the *snmf* function from the LEA package, to identify outlier loci based on *F*_*ST*_ only (François et al., 2016). As above, *p*-values were adjusted using the genomic inflation factor (λ) and false discovery rates were set using the Benjamini-Hochberg algorithm. Full R scripts of LFMM and genome scans for selection can be found in the supporting information of François et al. (2016) and the LEA website (http://membres-timc.imag.fr/Olivier.Francois/LEA/index.htm). To evaluate if adaptive signals differed between species we used a Chi-square test to compare the number of contigs containing candidate SNPs in relation to all the tested contigs.

In order to search for the proteins coded by the genes contained in the flanking regions of our candidate SNPs, contig sequences containing candidate loci were first submitted to the EMBOSS Transeq (http://www.ebi.ac.uk/Tools/st/emboss_transeq/) to obtain its corresponding protein sequences. We used all six frames with standard code (codon table), regions (start-end), trimming (yes), and reverse(no). We then ran a functional analysis using InterPro (https://www.ebi.ac.uk/interpro/; interproscan.sh -dp –appl PfamA,TIGRFAM,PRINTS,PrositePatterns,Gene3d –goterms –pathways -f tsv -o MySequences.tsv -i MySequences.faa), searching for go terms and pathways along the respective annotation databases (Interpro, Pfam, Tigrfam, Prints, PrositePattern and Gene3d).

## Results

### Genetic diversity and population structure

**Figure 2:**
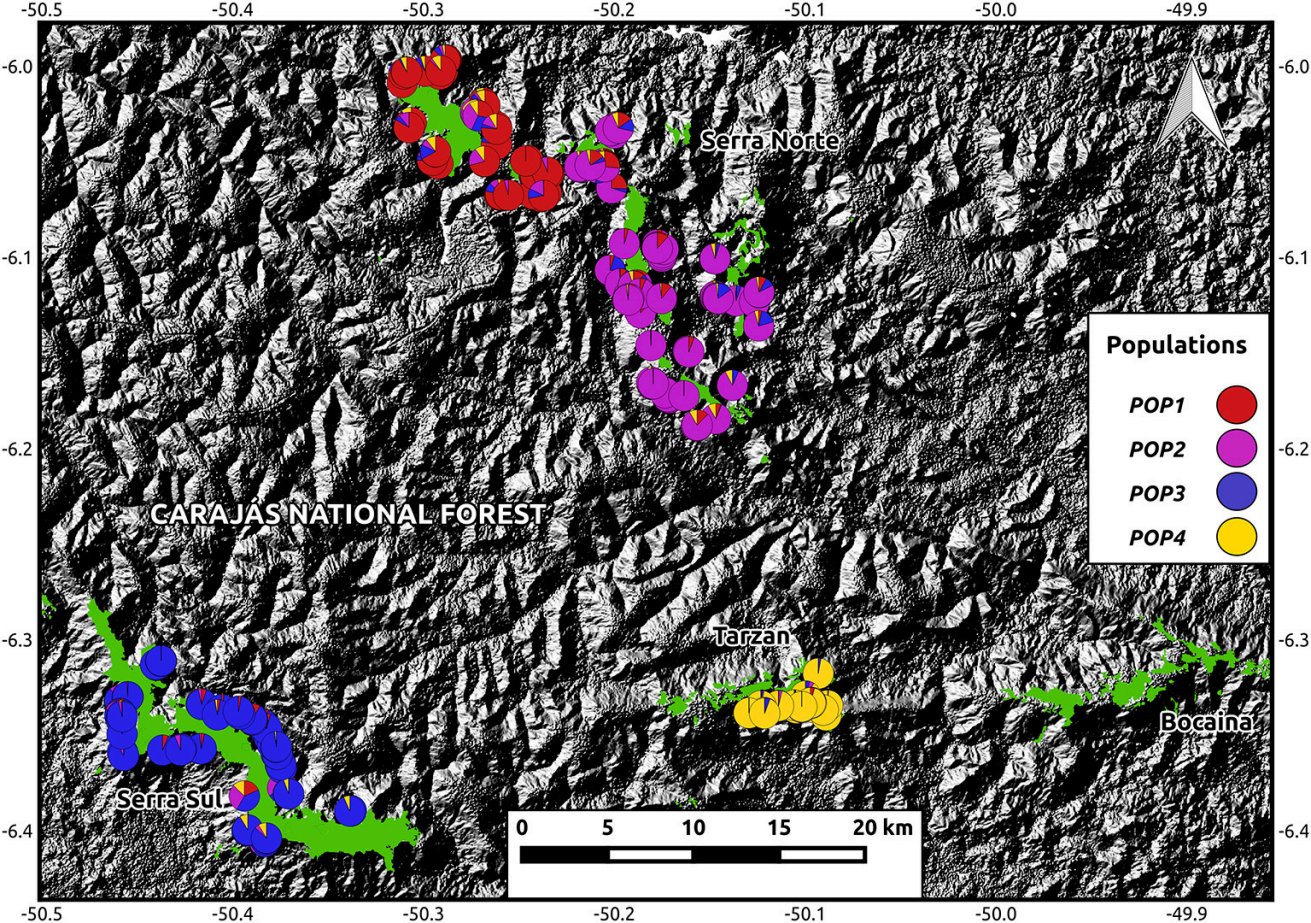
Map showing *I. maurandioides* assignments to four genetic clusters against an elevation map (from USGS Earth Explorer). Pie charts represent ancestry coefficients determined using the LEA package. Montane savanna highlands (from Souza-Filho et al., 2016) are shown in green. Coordinates are shown in decimal degrees.

A total of 41,881 SNPs were identified in *I. calvacantei* and 30,103 in *I. maurandioides*. After filtering these datasets (for quality, depth, LD, HWE, and candidate adaptive loci) we obtained sets of neutral markers containing 14,145 and 7,259 loci respectively. While heterozygosity and inbreeding levels were similar between *I. cavalcantei* and the analyzed populations of *I. maurandioides*, nucleotide diversity, Tajima's D and effective population size were higher in *I. cavalcantei* (Table 1). Two genetic clustering approaches indicated the presence of one population in *I. cavalcantei* and four populations in *I. maurandioides* (Figure 2, Figure S4, Data Sheet 3).

**Table 1:** Genetic diversity measures for *I. cavalcantei* and *I. maurandioides*.

**Species**	**Population**	**N**	***H_*E*_*(CI)**	***F* (CI)**	**π (CI)**	***D* (CI)**	***N_*e*_* (CI)**
*I. cavalcantei*	Pop1	122	0.18 (0.18/0.18)	0.12 (0.09/0.15)	0.18 (0.18/0.19)	0.16 (0.14/ 0.18)	855.60 (845.90/865.60)^a^
*I. maurandioides*	Pop1	79	0.17 (0.17/0.17)	0.07 (0.02/0.11)	0.13 (0.13/0.13)	0.05 (0.01/0.08)	182.80 (180.70/184.80)
	Pop2	63	0.18 (0.18/0.18)	0.01 (−0.01/0.04)	0.15 (0.15/0.15)	0.02 (−0.004/0.05)	224.20 (221.20/227.20)
	Pop3	90	0.18 (0.18/0.18)	0.03 (−0.02/0.08)	0.14 (0.14/0.15)	−0.02 (−0.05/0.003)	129.20 (128.30/130.00)
	Pop4	22	0.23 (0.23/0.23)	0.01 (−0.05/0.07)	0.13 (0.12/0.13)	−0.01 (−0.03/0.02)	222.80 (210.70/236.40)

*In the later species genetic diversity measures are grouped by genetic clusters. Sample sizes (N) are shown followed by mean expected heterozygosity (HE), mean inbreeding coefficient (F), mean per-site nucleotide diversity (π), Tajima's D (D) and effective population size (Ne). All estimates are shown along 95% confidence intervals (CI)*.

a *A similar estimate was found when analyzing a random sample of 22 I. cavalcantei individuals: N_e_ = 901.10 (834.80/978.70)*.

### Landscape genetic analyses

**Figure 3:**
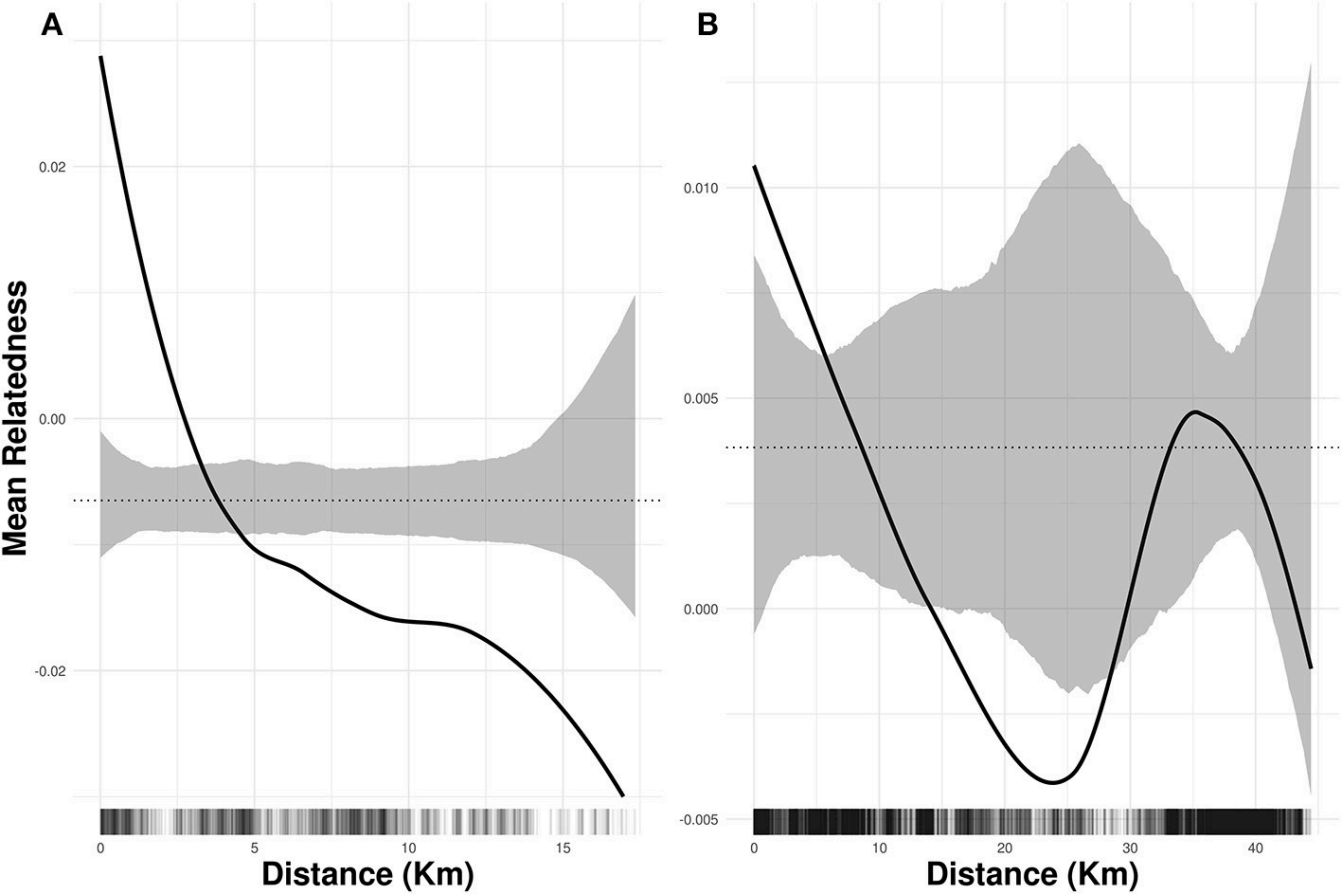
Spatial autocorrelation in genetic relatedness for *I. cavalcantei* **(A)** and *I. maurandioides* **(B)**. The black solid lines are the LOESS fit to the observed relatedness, while the grey shaded regions are 95% confidence bounds around the null expectation (black dotted lines). Short vertical lines at the bottom of each figure are observed pairwise distances.

We did not find evidence that pre-mining highland area and isolation were associated with current levels of heterozygosity and inbreeding (Table S3), and both species showed weak but significant genetic spatial autocorrelation for up to 5 km (Figure 3). The best models of isolation by landscape resistance (IBR) for *I. cavalcantei* included precipitation, temperature, terrain roughness, and land cover, but only these last two predictors significantly improved the model's log-likelihood (Table 2). Relatedness decreased with lower terrain roughness and higher land cover resistance (Tables 3, Figure S5). On the other hand, the best IBR models for *I. maurandioides* included precipitation, temperature, terrain roughness and land cover, but land cover was the only predictor that did not significantly improved the model's log-likelihood (Table 2). In this species relatedness decreased with lower precipitation, lower temperatures and higher terrain roughness (Table 3). Interestingly, univariate land cover models from all the tested years (1994, 2004, and 2013) were found to influence patterns of genetic relatedness in *I. maurandioides* but not in *I. cavalcantei*, where only land cover resistance distances from year 1994 were included in the set of best-fitting models (Table S4). The best land cover models for both species consistently revealed that forested and mining areas impose a higher resistance to gene flow than montane savanna areas (Table S5).

**Table 2:** Model selection summary showing the best MLPE models (ΔAICc ≤ 2) for each species.

**Species**	**Model**	**logLikelihood**	**AICc**	**ΔAICc**	**Weight**
*I.cavalcantei*	Precipitation + Inverse roughness^***^ + Land cover 1994 (Low montane savanna resistance)^***^	14847.54	−29683.10	0.00	0.53
	Temperature + Inverse roughness^***^ + Land cover 1994 (Low montane savanna resistance)^***^	14847.40	−29682.80	0.28	0.46
*I.maurandioides*	Inverse precipitation^***^ + Temperature^***^ + Roughness^***^ + Land cover 2013 (Low montane savanna resistance)	36747.27	−73480.50	0.00	0.86

All models contained inter-individual genetic relatedness as response variable and the different resistance distances as predictors. Likelihood Ratio Test (LRT) were performed to assess if each predictor variable significantly improved the model's log-likelihood (significance levels are highlighted with: ^*^p < 0.05; ^**^p < 0.01; and

*** *p < 0.001)*.

**Table 3:** Summary statistics of the best MLPE models (ΔAICc ≤ 2) for each species.

**Species**	**Predictor**	**Estimate**	***df***	***SE***	***t-*value**	***p-value***
*I. cavalcantei*	Precipitation	−4.82 × 10^−6^	7381	2.81 × 10^−6^	1.72	0.09
	Temperature	−3.71 × 10^−5^	7381	2.27 × 10^−5^	1.63	0.10
	Inverse roughness	−1.83 × 10^−4^	7381	3.50 × 10^−5^	5.24	<0.001
	Land cover 1994 (Low montane savanna resistance)	−8.62 × 10^−2^	7381	9.80 × 10^−3^	8.79	<0.001
*I. maurandioides*	Inverse precipitation	−3.41 × 10^−4^	32131	5.76 × 10^−5^	−5.93	<0.001
	Temperature	6.91 × 10^−5^	32131	1.49 × 10^−5^	4.64	<0.001
	Roughness	−2.26 × 10^−4^	32131	6.80 × 10^−5^	−3.33	<0.001
	Land cover 2013 (Low montane savanna resistance)	1.58 × 10^−3^	32131	3.41 × 10^−3^	0.46	0.64

*Model-averaged parameter estimates for each predictor variable are shown followed by degrees of freedom (df) standard errors (SE), t-values and p-values*.

### Identification of putative adaptive loci

**Figure 4:**
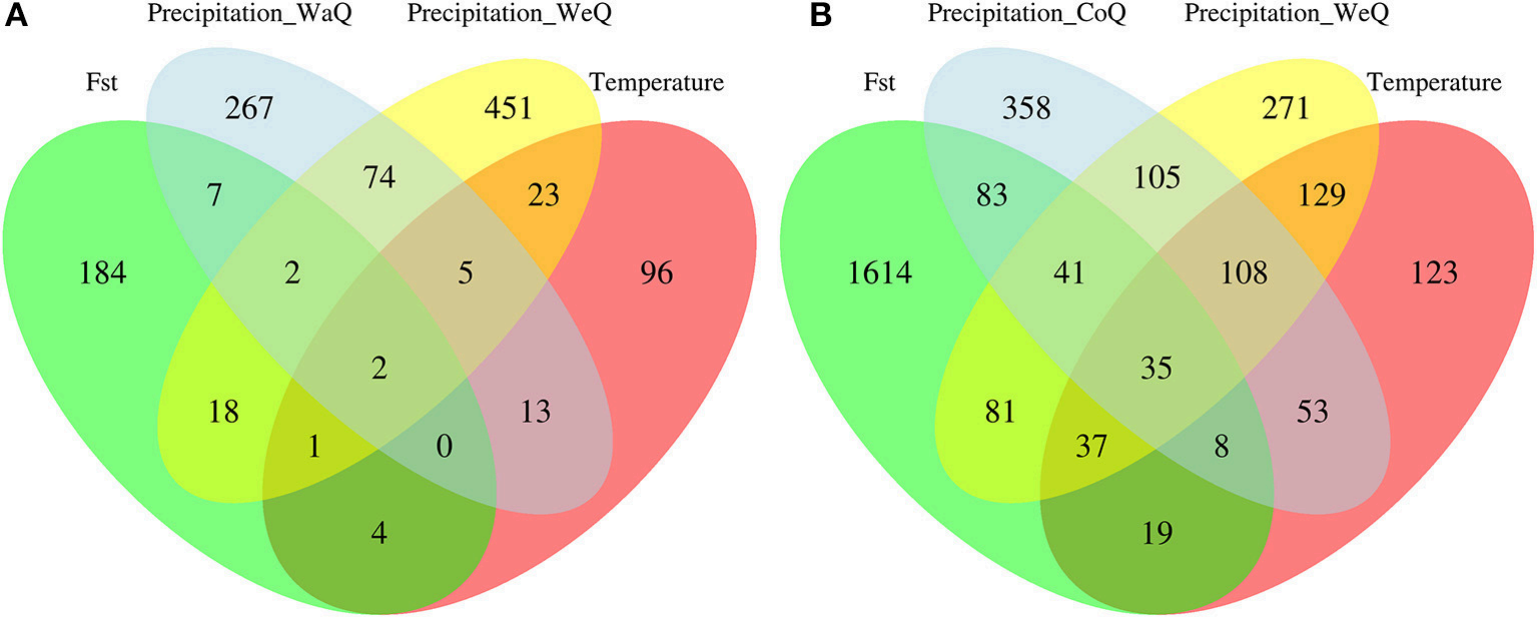
Venn diagram showing the intersection of sequences (contigs) containing candidate SNPs for *I. cavalcantei* **(A)** and *I. maurandioides* **(B)**. Putative adaptive loci were identified using environmental association tests and genome scans (*Fst* outlier tests), and the environmental variables used differed between species (*I. cavalcantei:* Minimum Temperature of Coldest Month, Precipitation of Warmest Quarter and Precipitation of Wettest Quarter; *I. maurandioides:* Minimum Temperature of Coldest Month, Precipitation of Coldest Quarter and Precipitation of Wettest Quarter).

We found 2,733 candidate SNPs out of the 34,102 that remained after filtering for depth and quality in *I. cavalcantei*, wereas we detected 5,238 candidate SNPs out of 23,181 analyzed SNPs in *I. maurandioides* (Table 4, Figure S6). We identified a total of 1,147 contigs containing putative adaptive SNPs out of 9,625 analyzed contig sequences in *I. cavalcantei* (11.92%), and 3,065 contigs containing candidate SNPs out of 9,143 analyzed contig sequences in *I. maurandioides* (33.52%; Chi-square test: *X*^2^ = 798.19, *p* < 0.001; Table 4, Figure S6). Within each species, some loci were identified with more than one statistical approach or were found associated with different environmental variables (Table S6, Figure 4). Only a subset of the identified putative adaptive genes contained InterPro annotations (40 in *I. cavalcantei* and 131 in *I. maurandioides*; Data Sheets 4, 5). Interestingly, we found annotated genes shared between both species that were associated with the same environmental variables (Table S7).

**Table 4:** Summary of the number of adaptive signals detected using environmental association and *F*_*ST*_outlier tests.

**Species**	**Signal type**	**Total analyzed**	**Total under selection**	**Environmental association tests^a^**			**Fst outlier tests**
				**Temperature**	**Precipitation (WaQ/CoQ)**	**Precipitation(WeQ)**	
*I. cavalcantei*	SNPs	34,102	2,733	327 (225)	904 (729)	1,513 (1,285)	262 (238)
	Contigs	9,625	1,147	144 (96)	370 (267)	576 (451)	218 (184)
*I. maurandioides*	SNPs	23,181	5,238	1,018 (331)	1,533 (841)	1,513 (642)	2,622 (2,331)
	Contigs	9,143	3,065	512 (123)	791 (358)	807 (271)	1,918 (1,614)

*Both the number of candidate SNPs and the number of contigs (RAD tags) containing candidate SNPs are presented for each methodological approach followed by the number of independent (non-overlapping) detections in parentheses*.

a *The tested environmental variables differed between species. I. cavalcantei: Minimum Temperature of Coldest Month (Temperature), Precipitation of Warmest Quarter (Precipitation WaQ) and Precipitation of Wettest Quarter (Precipitation WeQ); I. maurandioides: Minimum Temperature of Coldest Month (Temperature), Precipitation of Coldest Quarter (Precipitation CoQ) and Precipitation of Wettest Quarter (Precipitation WeQ)*.

## Discussion

Employing RAD sequencing to genotype thousands of genetic markers in two congeneric and sympatric morning glories from Amazonian savannas, we were able to compare genetic diversity, isolation by landscape resistance, and patterns of local adaptation in species belonging to different IUCN Red List Categories. Our results reveal higher genetic diversity and lower genetic differentiation in the endangered *I. cavalcantei* compared to the analyzed populations of the least concern *I. maurandioides*. Terrain roughness influenced gene flow in both species and temperature and precipitation were meaningful predictors of genetic relatedness patterns in *I. maurandioides*, whereas resistance to gene flow associated with forested and mining areas in existence two decades ago was found to influence IBR patterns in the narrowly distributed *I. cavalvantei*. Finally, we identified a set of putative adaptive genes associated to the same environmental variables in both species.

Contrary to our initial prediction, we found higher genetic diversity and lower spatial genetic structure in the narrow-endemic species. For instance, we found a larger *N*_*e*_ in *I. cavalcantei* than in *I. maurandioides* or *I. purpurea*, another widespread morning glory (Alvarado-Serrano et al., 2017), revealing that extent of occurrence (IUCN Red List Criterion B) is not a good predictor of genetic diversity in these plants (Frankham, 1996). Our large *N*_*e*_ estimate does not seem to be a sample size artifact (Table 1), and suggests the actual *I. cavalcantei* population is above the *N*_*e*_ threshold of 500 required to maintain long-term evolutionary potential (Jamieson and Allendorf, 2012). Although confidence intervals for inbreeding coefficients overlapped between species, our results reveal significant inbreeding for *I. cavalcantei* and one population of *I. maurandioides* (were 95% CI did not contain zero). While the panmictic population of *I. cavalcantei* occurs both in preserved and degraded montane savanna highlands (Figure 1), the one *I. maurandioides* population exposed to mining activities (Pop 2 in Figure 2) did not show higher inbreeding than any of the other three undisturbed populations (Table 1). Mining activities thus appear unrelated to observed inbreeding. Likewise, historical highland area and highland isolation were not found to be associated with inbreeding levels (Table S3). Observed inbreeding thus appears to be the result of some level of selfing (Leimu et al., 2006; Duncan and Rausher, 2013).

Two genetic clustering approaches identified the presence of a single panmictic population in *I. cavalcantei* (Figure S4), indicating relatively high levels of gene flow in the recent past, throughout its occurrence range. Interestingly, we found a small yet positive value of Tajima's D in *I. cavalcantei* (Table 1), which suggests the incidence of balancing selection or an ongoing population contraction (Simonsen et al., 1995), the later probably related to the expansion of mining areas (Figure S2). In contrast, we detected four spatially distinct populations in the more widely distributed *I. maurandioides*, two of which occurred within *I. cavalcantei*'s occurrence area (Figure 2, Data Sheet 3). Since founder effects result in higher spatial genetic structure and smaller effective population sizes (Booy et al., 2000; Allendorf et al., 2012), the recent colonization of our study region by few individuals of *I. maurandioides* could explain the observed patterns of population structure and genetic diversity. The fact that our study region is located at the northern edge of *I. maurandioides* distribution range (see Data Sheet 1), indeed hints at a possible recent colonization. However, we did not find negative Tajima's *D*-values, indicative of recent bottlenecks or population expansions (Simonsen et al., 1995). In fact Tajima's *D*-values overlapped with zero or were slightly positive in one population (Table 1), suggesting most populations were at mutation-drift equilibrium. Our results therefore fail to support the incidence of founder effects.

Life history differences influencing pollination and seed dispersal are known to be key predictors of gene flow and spatial genetic structure across plants (Vranckx et al., 2012; Gelmi-Candusso et al., 2017), so we believe these are likely to explain our observed patterns of genetic structure. Although our dataset does not allow disentangling the relative contribution of pollen-movement and seed dispersal to gene flow, wind-mediated seed dispersal is likely to be more restricted, as forest surrounding the montanne savanna highlands constitute natural wind barriers. For instance, a recent work found strong spatial genetic structure when analyzing seven Chloroplast DNA loci in *I. cavalcantei* (Babiychuk et al., 2017), thus suggesting restricted seed dispersal in this species. Additionally, the significant (albeit low) spatial autocorrelation of genotypes found in both species (Figure 3) indicates the existence of patches of related individuals from limited seed donors (Davies et al., 2010). This fine-space genetic structure, however, disappears at larger spatial scales, suggesting a role of pollen movement in maintaining gene flow across different montanne savanna highlands (Sork and Smouse, 2006). The observed differences in spatial genetic structure between both species thus appear to be related to the dispersal capabilities of their pollinators (Sork and Smouse, 2006), and support earlier findings showing higher genetic structure in bee-pollinated plants compared to hummingbird-pollinated species (Kramer et al., 2011).

The IBR patterns detected here are thus likely to reflect the influence of local landscape on pollinator-mediated pollen transfer. Terrain roughness, for instance, seems to have facilitated humming-bird mediated pollination but hindered insect-mediated pollen transfer (Table 3). Indeed topographic complexity has been found to influence gene flow in other organisms (Murphy et al., 2010; Noguerales et al., 2016), and elevation has been associated to IBR in other tropical plants (Castilla et al., 2016). On the other hand, climatic factors (temperature and precipitation) were only found to influence gene flow in *I. maurandioides*, indicating they could have affected bee dispersal (Jaffé et al., 2016) or caused plant phenological differences (Kramer et al., 2011).

Two lines of reasoning suggest our genetic diversity estimates mainly reflect gene flow taking place in the recent past. First, our estimates were based on neutral and independent nuclear loci. Second, heterozygosity and inbreeding were unaffected by historical highland area or highland isolation (Table S3). Interestingly, we found that only the oldest land cover map (1994) was able to explain the observed genetic relatedness patterns in *I. cavalcantei*, while all maps (1994–2013) were equally informative in the case of *I. maurandioides* (Table S4). This result was expected, given that generation times are much longer in the former species, and indicate a longevity dependent time-lag between land cover changes and genetic differentiation patterns (Landguth et al., 2010). Thus, our *I. cavalcantei* data do not allow us assessing restrictions to gene flow due to land cover changes that occurred after 1994.

In spite of these temporal limitations, land cover significantly influenced gene flow in the narrow-endemic but not in the broadly distributed species, as we initially predicted (Table 3). Specifically, forested and mining areas were found to impose a higher resistance to gene flow than montane savanna areas (Table S5), a result that confirms the importance of this unique ecosystem as the exclusive habitat of this morning glory (Skirycz et al., 2014; Viana et al., 2016). Forested and mining areas thus appear to hinder hummingbird-mediated pollination, suggesting an effect of habitat (montane savanna) fragmentation (Hadley et al., 2014). This effect, however, did not influence overall genetic structure, indicating that a substantial level of gene flow is still maintained across forested areas. The impact of mining on population-wide genetic structure is nevertheless more difficult to assess, as such an effect would only appear after several generations exposed to mining (Landguth et al., 2010).

Our results support our prediction of weaker adaptive signals (a lower proportion of candidate loci) in the narrow-endemic species, thereby suggesting that stronger genetic drift is slowing adaptive responses in *I. cavalcantei* (Allendorf et al., 2012). Although local climatic variability was limited, due to the resolution of bioclimatic variables (see Table S8), we believe that our environmental association tests were able to identify true adaptive signals. For instance, our environmental association tests can be considered conservative, since they accounted for false discovery rates and showed genomic inflation factors below 1 (Table S6; François et al., 2016). Moreover, LFMM results are robust to deviations from the underlying demographic structure, as candidate loci were intersected across different *k*-values. On the other hand, *F*_*ST*_ outlier tests showed genomic inflation factors above 1 (Table S6; François et al., 2016), indicating these were more liberal tests. We therefore caution about a higher incidence of Type I errors in our *F*_*ST*_ outlier tests, and highlight the need for follow-up studies involving different analytical approaches or functional validation. Likewise, we note that many other genes occurring in the flanking regions of our candidate SNPs could be responsible for the detected adaptive signals, so fine genomic mapping is necessary to confirm these genes.

In many cases we did not find any matches for our translated proteins, or found matches with uncharacterized proteins. This explains the lower number of putative adaptive proteins identified compared to the initial number of candidate loci. One of these proteins (reverse transcriptase, RNA-dependent DNA polymerase) was found associated to all environmental variables and was also identified through *F*_*ST*_ outlier tests in both species (Table S7). This protein indicates the presence of retrotransposons, as does ribonuclease H-like domain, retroviral aspartyl protease, retrotransposon gag domain, and integrase. Retrotransposons are abundant and ancient components of plant genomes associated to stress response (Grandbastien, 1998). Indeed reverse transcriptase acts as key initiator of defense responses in flowering plants (Sarris et al., 2016), while Leucine-rich repeat domain, CAP domain proteins, knottin and defensin plant proteins are also involved in plant defenses against pathogens (Gibbs et al., 2008; Benko-Iseppon et al., 2010; Tavormina et al., 2015). On the other hand, WPP domain and CCHC zinc finger proteins are involved in plant cell division (Patel et al., 2004; Clay and Nelson, 2005), and death domain proteins in apoptotic signaling (Coll et al., 2011). Finally, ClpP/crotonases, hydrolases, esterases, helicases and pectinesterases carry out a wide range of metabolic functions (Holden et al., 2001; Xu et al., 2004). Interestingly, in both species we found proteins identified using *F*_*ST*_ outlier tests that did not overlap with those found by environmental association tests (Table 4). Our results thus suggest that these proteins are signaling general responses, unrelated to environmental variations. In sum, most of the identified putative proteins appear to be related to pathogen resistance and physiological adaptations to life in the extreme environments of montane savannas (Skirycz et al., 2014; Silveira et al., 2015; Viana et al., 2016). These potential adaptations are relevant to conservation and restoration initiatives seeking the protection of adaptive genetic diversity or the introduction of individuals adapted to adverse environmental conditions into degraded areas (Shryock et al., 2017).

Most conservation geneticists have used the 50/500 rule to define minimum viable populations (Jamieson and Allendorf, 2012; Franklin et al., 2014): An *N*_*e*_ of 50 is deemed necessary to avoid short-term inbreeding depression, while an *N*_*e*_ of 500 is recommended to maintain long-term evolutionary potential. More recently, Willoughby et al. (2015) presented a novel approach for identifying species of conservation need by estimating the expected loss of genetic diversity. Their method, however, requires access to a large database of heterozygosity values from related species in order to establish a cut off value. Since we do not have access to such a database we cannot implement this novel approach to re-assess IUCN Red List Categories. Instead, we base our conservation recommendations on the 50/500 rule. In the case of the narrow-endemic *I. cavalcantei*, which showed comparatively higher genetic diversity and high levels of gene flow across its entire occurrence range, conservation efforts should be oriented to preserve long-term evolutionary potential (*N*_*e*_ > 500). On the other hand, precautionary conservation measures should aim at maintaining *N*_*e*_ > 50 within each population to avoid short term inbreeding depression in *I. maurandioides*. Although land cover was found to influence gene flow in the the narrow-endemic species, overall population panmixia indicates that a substantial level of genetic connectivity is maintained across the fragmented habitat of *I. cavalcantei*. Additionally, we identified possible adaptations for life in Amazonian savannas, which could inform future conservation and restoration programs.

Our study exemplifies how landscape genomic tools can be applied to assess the conservation status, susceptibility to global change, and adaptive responses of imperiled biodiversity. More specifically, our genomic assessment revealed unexpected patterns of genetic diversity, spatial genetic structure and gene flow in tropical plant species belonging to different IUCN Red List Categories. Given the level of resolution of our genetic diversity and gene flow estimates, our findings emphasize that the IUCN Red List Criteria approach does not systematically prioritize the conservation of species with low genetic diversity (Rivers et al., 2014; Willoughby et al., 2015) or highest susceptibility to habitat loss and fragmentation, and call for the inclusion of genetic information into conservation assessments (an issue currently evaluated by the IUCN Conservation Genetics Specialist Group: http://www.cgsg.uni-freiburg.de/). We are well aware this will be no easy endeavor, as funding limitations, country-specific conservation policies and communication barriers between scientists and practitioners are among the issues to overcome (Hoban et al., 2013; Bowman et al., 2016; Pierson et al., 2016; Taylor et al., 2017), but would like to stress out that the demographic parameters employed to assess conservation status two decades ago have remained unchanged in the age of next-generation-sequencing and meta-genomics.

## Author contributions

RJ: conceived, designed and coordinated the project; RJ and VI-F: initiated the project; RJ, ÉL, and AG: coordinated the field work and sampling; ÉL, NC, WM, and SV: performed laboratory work; RJ, ÉL, NP, RA, WM, AS, and PS-F: performed the data analysis. The first draft of the paper was written by ÉL with input from RJ. All authors contributed to discussing the results and editing the paper.

### Conflict of interest statement

The authors declare that the research was conducted in the absence of any commercial or financial relationships that could be construed as a potential conflict of interest.
