# Supplementary material for: Landscape Genomic Conservation Assessment of a Narrow-Endemic and a Widespread Morning Glory From Amazonian Savannas
Source: Front Plant Sci. 2018 May 7;9:532. doi: 10.3389/fpls.2018.00532 (PMC5949356; doi:10.3389/fpls.2018.00532)
Supplement: Supplementary file 1 [file Table_1.pdf]

Table S1: Genome size for the two study species estimated through flow cytometry.

Species	N	2C DNA content (pg)		
		**Mean**	**SD**	**Min-Max**
I. cavalcantei [a]	31	2.46	0.10	2.22-2.61
I. maurandioides	7	2.21	0.20	2.06-2.58

[a] Data taken from (Babiychuk *et al.* 2017).
