# Supplementary material for: Landscape Genomic Conservation Assessment of a Narrow-Endemic and a Widespread Morning Glory From Amazonian Savannas
Source: Front Plant Sci. 2018 May 7;9:532. doi: 10.3389/fpls.2018.00532 (PMC5949356; doi:10.3389/fpls.2018.00532)
Supplement: Supplementary file 2 [file Table_2.pdf]

Table S2: Spatial data specifications.

**Product**	**Source**	**Format**	**Temporal extent**	**Spatial resolution**
Land cover [a]	(Souza-Filho *et al.* 2016)	Vector (ESRI shapefile)	1984, 1994, 2004 and 2013	1 arc-second (~30 m) [a]
Elevation (SRTM) [b]	https://earthexplorer.usgs.gov/	Raster (GeoTiff)	2014	1 arc-second (~30 m)
Bioclimatic variables	http://www.worldclim.org/	Raster (GeoTiff)	1960-1990	30 arc-seconds (~1 km^2)

[a] Land cover maps were rasterized using a resolution of 3 arc-seconds (~90 m).
[b] Terrain roughness was calculated from this elevation map using the Terrain Analysis plug-in in QGIS V2.14.
