# Supplementary material for: Landscape Genomic Conservation Assessment of a Narrow-Endemic and a Widespread Morning Glory From Amazonian Savannas
Source: Front Plant Sci. 2018 May 7;9:532. doi: 10.3389/fpls.2018.00532 (PMC5949356; doi:10.3389/fpls.2018.00532)
Supplement: Supplementary file 3 [file Table_3.pdf]

Table S3: Effect of pre-mining highland area and isolation on expected heterozygosity (H_E) and inbreeding (F). The table shows X^2 values from likelihood ratio tests followed by p-values in parentheses.

**Species** [a]	**Variable**	**Highland area**	**Highland isolation**
I. cavalcantei	H_E	1.63 (p = 0.20)	1.84 (p = 0.17)
	F	0.07 (p = 0.79)	1.88 (p = 0.17)
I. maurandioides	H_E	0.79 (p = 0.37)	0.76 (p = 0.38)
	F	1.87 (p = 0.17)	2.44 (p = 0.12)

[a] While both highland area and isolation were included as predictors in *I. cavalcantei* models (because they were uncorrelated: r = 0.48), simple models were ran for *I. maurandioides* (because both variables were correlated: r = 0.84).
