# Supplementary material for: Landscape Genomic Conservation Assessment of a Narrow-Endemic and a Widespread Morning Glory From Amazonian Savannas
Source: Front Plant Sci. 2018 May 7;9:532. doi: 10.3389/fpls.2018.00532 (PMC5949356; doi:10.3389/fpls.2018.00532)
Supplement: Supplementary file 4 [file Table_4.PDF]

Table S4: Model selection summary showing the best MLPE land cover models (ΔAICc ≤ 2) for each species. All models contained inter-individual genetic relatedness as response variable and distances retrieved from the different land cover resistance surfaces as predictors (see methods for details).

**Species**	**Land cover model**	**logLikelihood**	**AICc**	**ΔAICc**	**Weight**
I.cavalcantei	Land cover 1994 (Low montane savanna resistance)	14823.77	-29639.50	0.00	0.97
I. maurandioides	Land cover 2013 (Low montane savanna resistance)	36723.17	-73438.30	0.00	0.30
	Land cover 2004 (Low montane savanna resistance)	36722.54	-73437.10	1.26	0.16
	Land cover 1994 (Low montane savanna resistance)	36722.34	-73436.70	1.66	0.13
