# Supplementary material for: Landscape Genomic Conservation Assessment of a Narrow-Endemic and a Widespread Morning Glory From Amazonian Savannas
Source: Front Plant Sci. 2018 May 7;9:532. doi: 10.3389/fpls.2018.00532 (PMC5949356; doi:10.3389/fpls.2018.00532)
Supplement: Supplementary file 5 [file Table_5.PDF]

Table S5: Summary statistics of the best MLPE land cover models. Parameter estimates are shown followed by degrees of freedom (df), standard errors (SE), *t*-values and *p*-values.

**Species**	**Predictor**	**Estimate**	**df**	**SE**	***t*-value**	***p*-value**
I.cavalcantei	Land cover 1994 (Low montane savanna resistance)	-1.50×10^{-1}	7381	3.72×10^{-3}	-40.27	<0.001
I. maurandioides	Land cover 2013 (Low montane savanna resistance)	-7.78×10^{-3}	32131	2.16×10^{-3}	-3.60	<0.001
	Land cover 2004 (Low montane savanna resistance)	-7.53×10^{-3}	32131	2.20×10^{-3}	-3.42	<0.001
	Land cover 1994 (Low montane savanna resistance)	-7.45×10^{-3}	32131	2.21×10^{-3}	-3.36	<0.001
