# Supplementary material for: Landscape Genomic Conservation Assessment of a Narrow-Endemic and a Widespread Morning Glory From Amazonian Savannas
Source: Front Plant Sci. 2018 May 7;9:532. doi: 10.3389/fpls.2018.00532 (PMC5949356; doi:10.3389/fpls.2018.00532)
Supplement: Supplementary file 6 [file Table_6.PDF]

Table S6: Genomic Inflation Factor (λ) used in environmental association tests (EAT) and genome scans (F_{ST} outlier tests).

**Species**	**Test**	**Number of populations (k)**	**Environmental variable** [a]	λ [b]
I. cavalcantei	EAT	1	1	0.56
		1	2	0.45
		1	3	0.62
		2	1	0.50
		2	2	0.40
		2	3	0.60
		3	1	0.52
		3	2	0.40
		3	3	0.62
	Genome scan	*2*	-	2.40
I. maurandioides	EAT	2	1	0.70
		2	2	0.60
		2	3	0.50
		3	1	0.70
		3	2	0.60
		3	3	0.50
		4	1	0.70
		4	2	0.60
		4	3	0.52
		5	1	0.65
		5	2	0.60
		5	3	0.50
		6	1	0.70
		6	2	0.60
		6	3	0.50
	Genome scan	*4*	-	7.00

[a] *I. cavalcantei:* 1 = Min Temperature of Coldest Month, 2 = Precipitation of Warmest Quarter and 3 = Precipitation of Wettest Quarter; *I. maurandioides:* 1 = Min Temperature of Coldest Month, 2 = Precipitation of Wettest Quarter and 3 = Precipitation of Coldest Quarter. [b] False discovery rates were set using the Benjamini-Hochberg algorithm and $q = 0.05$ in all cases.
