# Supplementary material for: Landscape Genomic Conservation Assessment of a Narrow-Endemic and a Widespread Morning Glory From Amazonian Savannas
Source: Front Plant Sci. 2018 May 7;9:532. doi: 10.3389/fpls.2018.00532 (PMC5949356; doi:10.3389/fpls.2018.00532)
Supplement: Supplementary file 7 [file Table_7.PDF]

Table S7: Annotated putative adaptive proteins shared between both species, identified using environmental association tests (EAT) and genome scans for selection (F_{ST} outlier tests).

Statistical approach	Climatic variable	Signature description
EAT	Min Temperature of Coldest Month	Reverse transcriptase, RNA-dependent DNA polymerase
		Ribonuclease H-like domain
		Retroviral aspartyl protease
		Leucine-rich repeat domain, L domain-like
	Precipitation of Wettest Quarter	Ribonuclease H-like domain
		Integrase, catalytic core
		Reverse transcriptase zinc-binding domain
		Reverse transcriptase, RNA-dependent DNA polymerase
		Retrotransposon gag domain
		Zinc finger, CCHC-type
		ClpP/crotonase-like domain
		Leucine-rich repeat domain, L domain-like
	Precipitation of Warmest/Coldest Quarter	Ribonuclease H-like domain
		Winged helix-turn-helix DNA-binding domain
		Reverse transcriptase, RNA-dependent DNA polymerase
		Zinc finger, CCHC-type
		P-loop containing nucleoside triphosphate hydrolase
Genome scan	-	NAD(P)-binding domain
		PC-Esterase
		Reverse transcriptase, RNA-dependent DNA polymerase
		DNA helicase Pif1-like
		Pectinesterase inhibitor domain
