# Supplementary material for: Landscape Genomic Conservation Assessment of a Narrow-Endemic and a Widespread Morning Glory From Amazonian Savannas
Source: Front Plant Sci. 2018 May 7;9:532. doi: 10.3389/fpls.2018.00532 (PMC5949356; doi:10.3389/fpls.2018.00532)
Supplement: Supplementary file 8 [file Table_8.PDF]

**Table S8**: Value ranges for environmental variables extracted from sample coordinates of the two study species. Environmental variables marked with * were used to run Environmental Association Tests (LFMM).

| Species | Environmental variable [a] | Min | Max |
|---|---|---|---|
| *I. cavalcantei* | BIO1 | 230 | 241 |
| | BIO2 | 111 | 112 |
| | BIO3 | 81 | 82 |
| | BIO4 | 304 | 379 |
| | BIO5 | 300 | 311 |
| | BIO6* | 164 | 176 |
| | BIO7 | 135 | 137 |
| | BIO8 | 228 | 238 |
| | BIO9 | 231 | 243 |
| | BIO10 | 233 | 245 |
| | BIO11 | 225 | 236 |
| | BIO12 | 1897 | 1934 |
| | BIO13 | 304 | 308 |
| | BIO14 | 21 | 22 |
| | BIO15 | 62 | 64 |
| | BIO16* | 843 | 857 |
| | BIO17 | 94 | 99 |
| | BIO18* | 136 | 253 |
| | BIO19 | 772 | 835 |
| | Elevation | 555 | 791 |
| *I. maurandioides* | BIO1 | 224 | 244 |
| | BIO2 | 111 | 116 |
| | BIO3 | 80 | 82 |
| | BIO4 | 290 | 379 |
| | BIO5 | 298 | 315 |
| | BIO6* | 155 | 179 |
| | BIO7 | 135 | 143 |
| | BIO8 | 222 | 242 |
| | BIO9 | 226 | 246 |
| | BIO10 | 228 | 248 |
| | BIO11 | 220 | 240 |
| | BIO12 | 1878 | 1953 |
| | BIO13 | 295 | 307 |
| | BIO14 | 20 | 23 |
| | BIO15 | 59 | 64 |
| | BIO16* | 817 | 857 |
| | BIO17 | 91 | 101 |
| | BIO18 | 136 | 286 |
| | BIO19* | 762 | 836 |
| | Elevation | 568 | 802 |

[a] BIO1 = Annual Mean Temperature; BIO2 = Mean Diurnal Range (Mean of monthly (max temp - min

temp)); BIO3 = Isothermality (BIO2/BIO7) (* 100); BIO4 = Temperature Seasonality (standard deviation *100); BIO5 = Max Temperature of Warmest Month; BIO6 = Min Temperature of Coldest Month; BIO7 = Temperature Annual Range (BIO5-BIO6); BIO8 = Mean Temperature of Wettest Quarter; BIO9 = Mean Temperature of Driest Quarter; BIO10 = Mean Temperature of Warmest Quarter; BIO11 = Mean Temperature of Coldest Quarter; BIO12 = Annual Precipitation; BIO13 = Precipitation of Wettest Month; BIO14 = Precipitation of Driest Month; BIO15 = Precipitation Seasonality (Coefficient of Variation); BIO16 = Precipitation of Wettest Quarter; BIO17 = Precipitation of Driest Quarter; BIO18 = Precipitation of Warmest Quarter; BIO19 = Precipitation of Coldest Quarter; Elevation = Digital Elevation (SRTM). See data sources in Table S2.
