# Supplementary material for: Landscape Genomic Conservation Assessment of a Narrow-Endemic and a Widespread Morning Glory From Amazonian Savannas
Source: Front Plant Sci. 2018 May 7;9:532. doi: 10.3389/fpls.2018.00532 (PMC5949356; doi:10.3389/fpls.2018.00532)
Supplement: Supplementary file 9 [file Image_1.PDF]

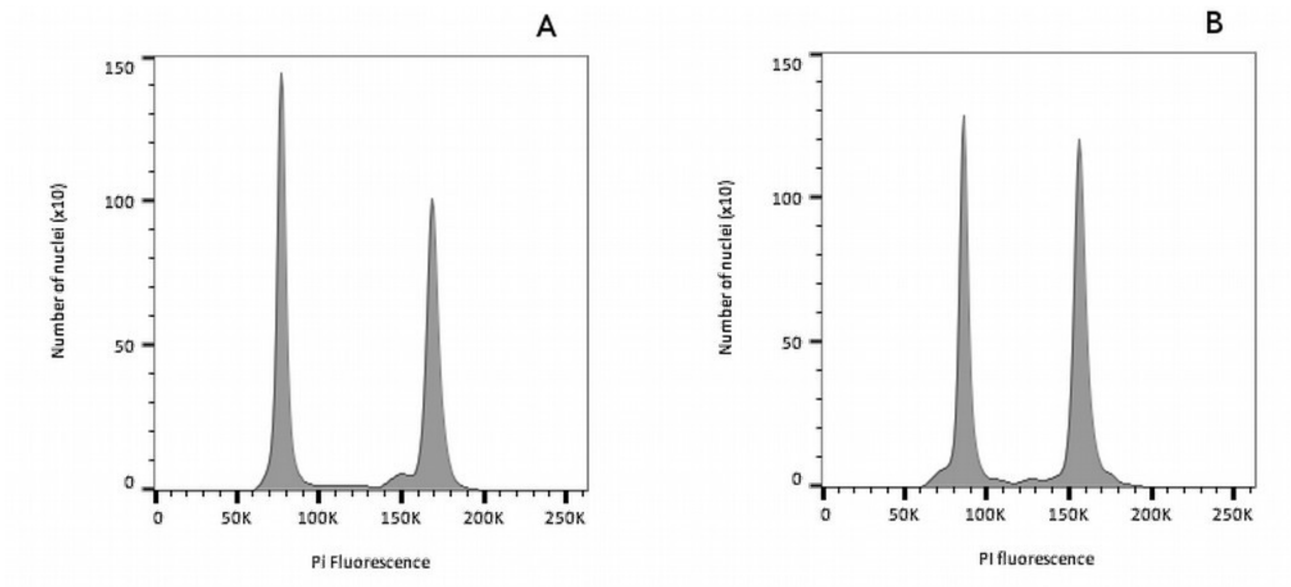

Figure S1: Histograms showing genome size estimates for *I. cavalcantei* (A) and *I. maurandioides* (B). In each figure the focus species (left hand peak) in shown next to a reference species (*Petroselinum crispum*, right hand peak). Data from *I. cavalcantei* was taken from (Babiychuk *et al.* 2017).
