# Supplementary material for: Landscape Genomic Conservation Assessment of a Narrow-Endemic and a Widespread Morning Glory From Amazonian Savannas
Source: Front Plant Sci. 2018 May 7;9:532. doi: 10.3389/fpls.2018.00532 (PMC5949356; doi:10.3389/fpls.2018.00532)
Supplement: Supplementary file 10 [file Image_2.PDF]

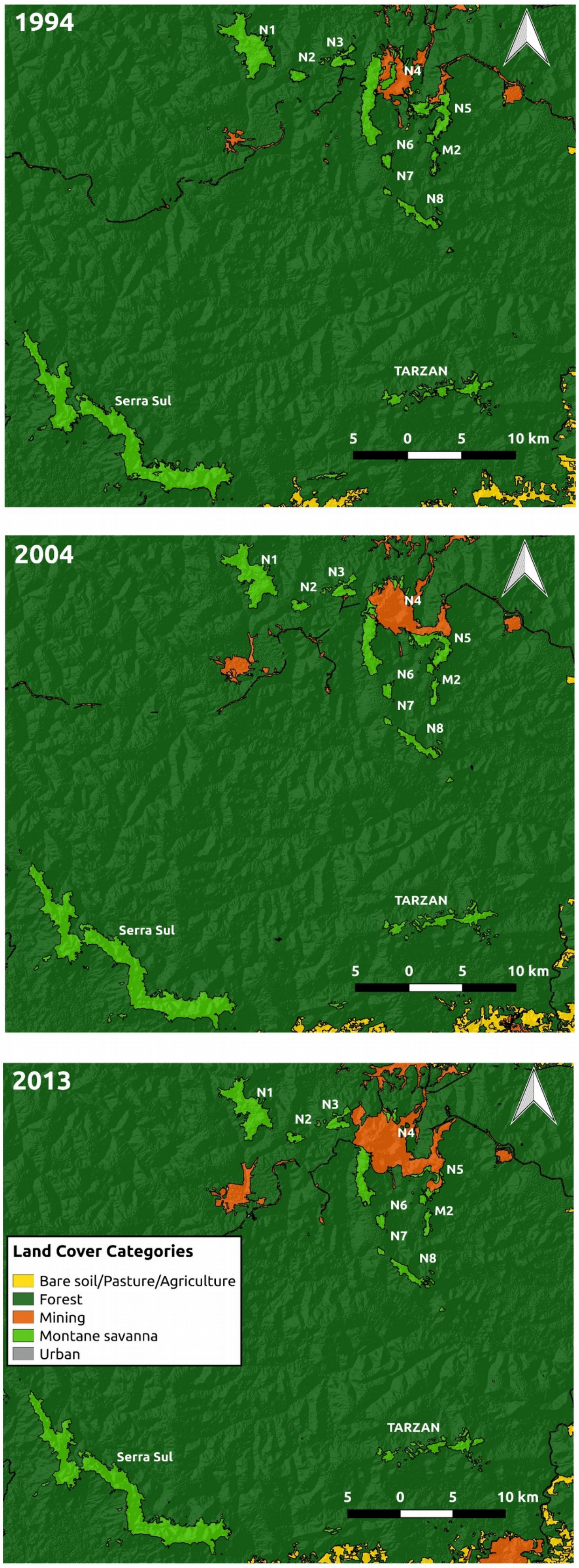

Figure S2: Land cover maps of the study region for years 1994, 2004 and 2013. An elevation (hill shade) map is shown overlaid with the land cover color maps.
