# Supplementary material for: Landscape Genomic Conservation Assessment of a Narrow-Endemic and a Widespread Morning Glory From Amazonian Savannas
Source: Front Plant Sci. 2018 May 7;9:532. doi: 10.3389/fpls.2018.00532 (PMC5949356; doi:10.3389/fpls.2018.00532)
Supplement: Supplementary file 11 [file Image_3.PDF]

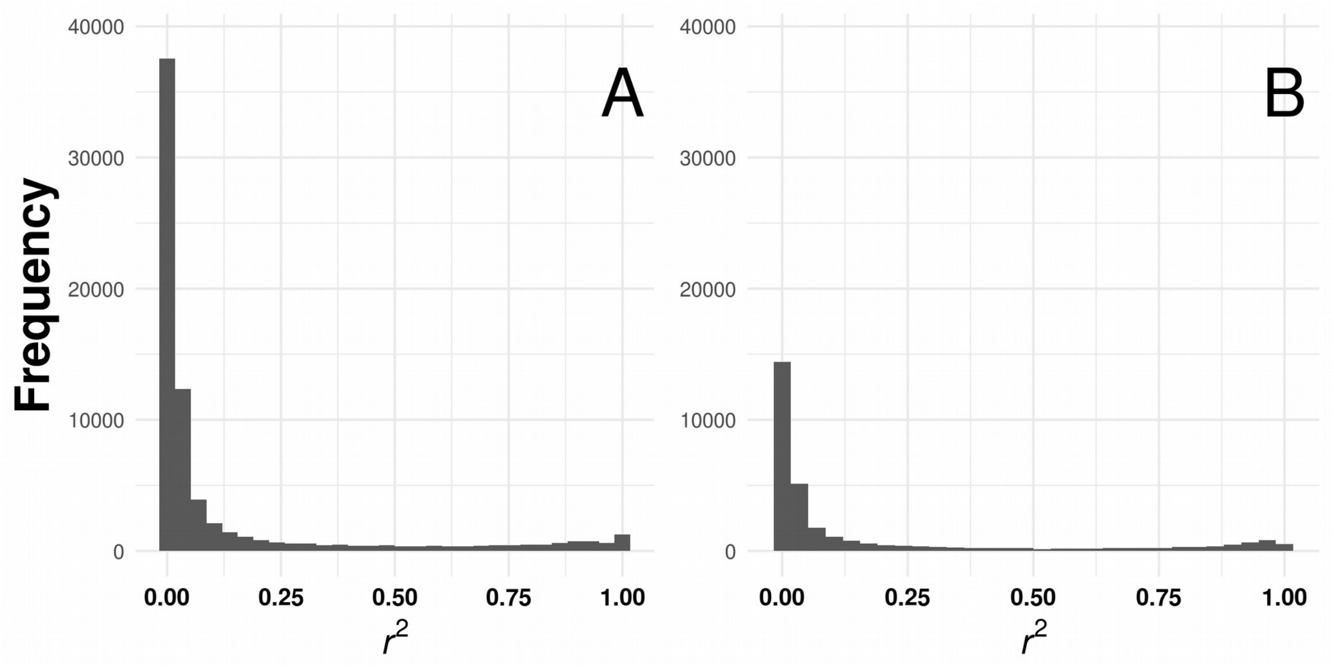

Figure S3: Frequency distribution of linkage disequilibrium within contigs in *I. cavalcantei* (A) and *I. maurandioides* (B).
