# Supplementary material for: Landscape Genomic Conservation Assessment of a Narrow-Endemic and a Widespread Morning Glory From Amazonian Savannas
Source: Front Plant Sci. 2018 May 7;9:532. doi: 10.3389/fpls.2018.00532 (PMC5949356; doi:10.3389/fpls.2018.00532)
Supplement: Supplementary file 12 [file Image_4.PDF]

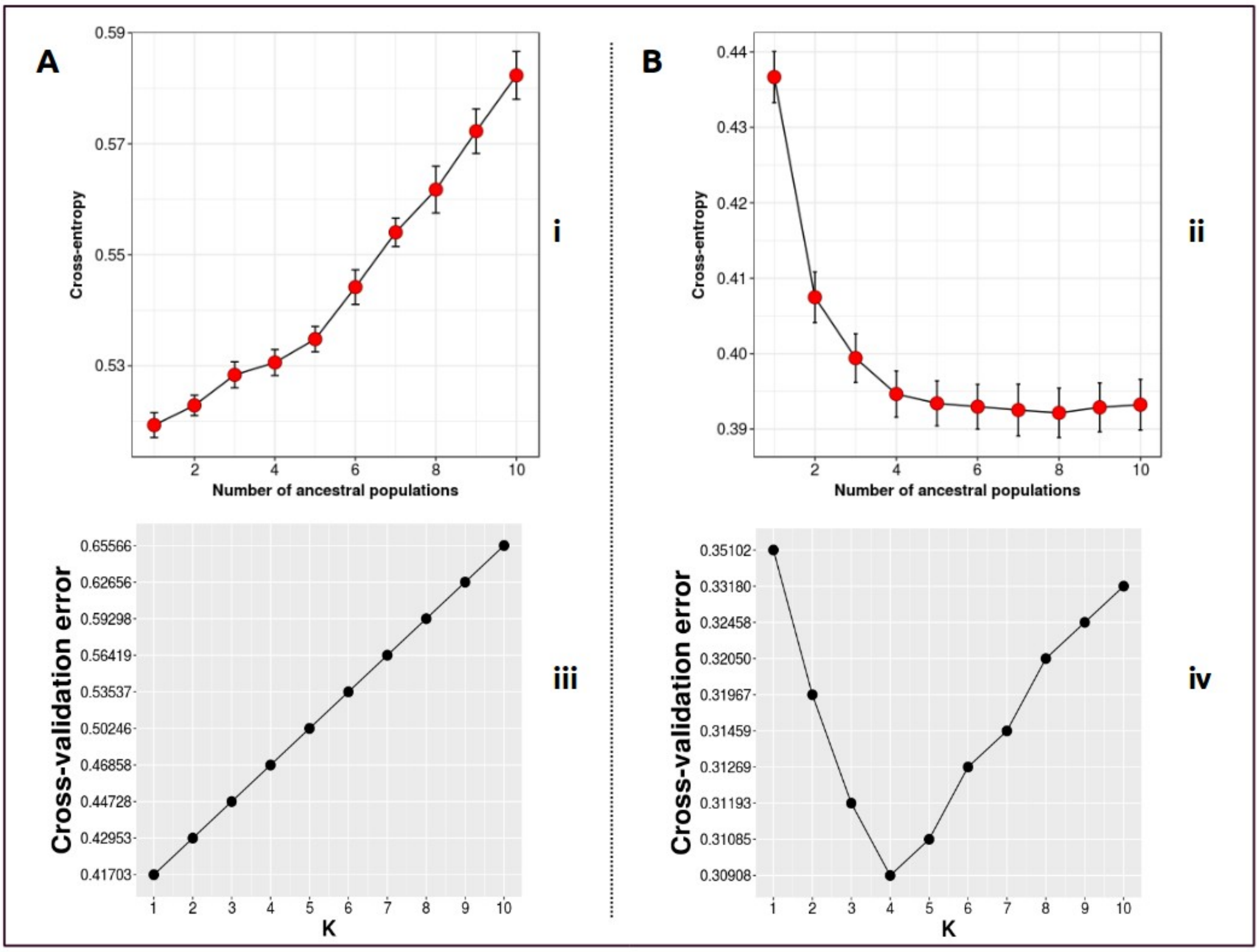

Figure S4: Plots showing the optimal number of genetic clusters (k) for *I. cavalcantei* (A) and *I. maurandioides* (B). Optimal k choice is based on mean ± sd cross-entropy (LEA, i and ii) and cross-validation errors (Admixture, iii and iv).
