# Supplementary material for: Landscape Genomic Conservation Assessment of a Narrow-Endemic and a Widespread Morning Glory From Amazonian Savannas
Source: Front Plant Sci. 2018 May 7;9:532. doi: 10.3389/fpls.2018.00532 (PMC5949356; doi:10.3389/fpls.2018.00532)
Supplement: Supplementary file 13 [file Image_5.PDF]

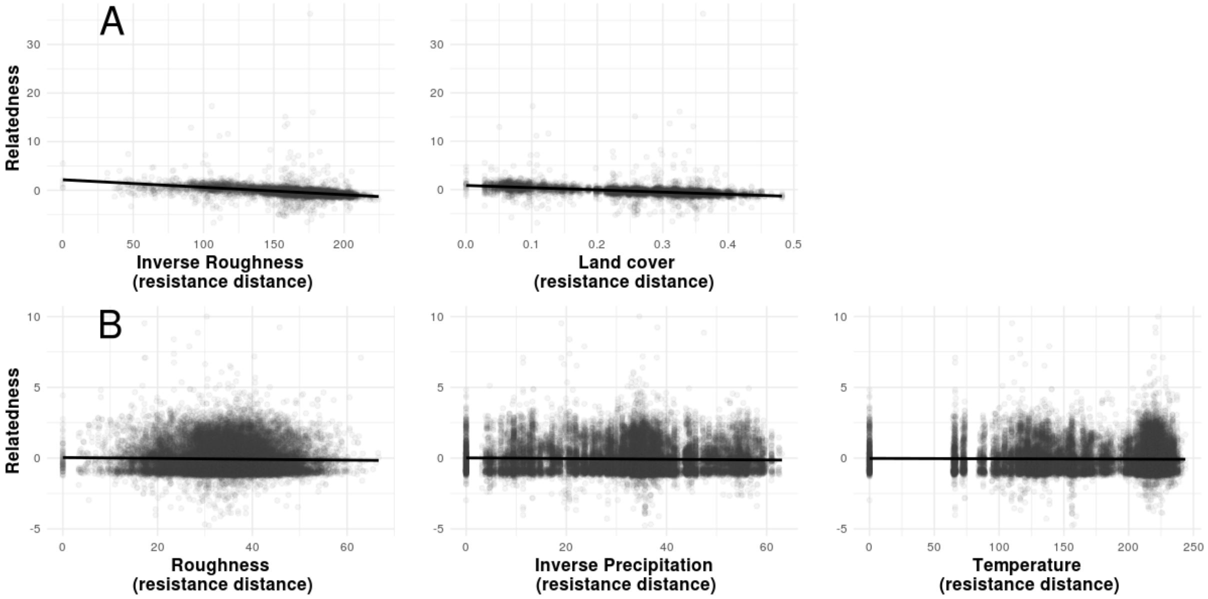

Figure S5: Relationship between inter-individual genetic relatedness and landscape resistance in *I. cavalcantei* (A) and *I. maurandioides* (B). Genetic relatedness is de-correlated for the MLPE correlation structure.
