# Supplementary material for: Landscape Genomic Conservation Assessment of a Narrow-Endemic and a Widespread Morning Glory From Amazonian Savannas
Source: Front Plant Sci. 2018 May 7;9:532. doi: 10.3389/fpls.2018.00532 (PMC5949356; doi:10.3389/fpls.2018.00532)
Supplement: Supplementary file 14 [file Image_6.PDF]

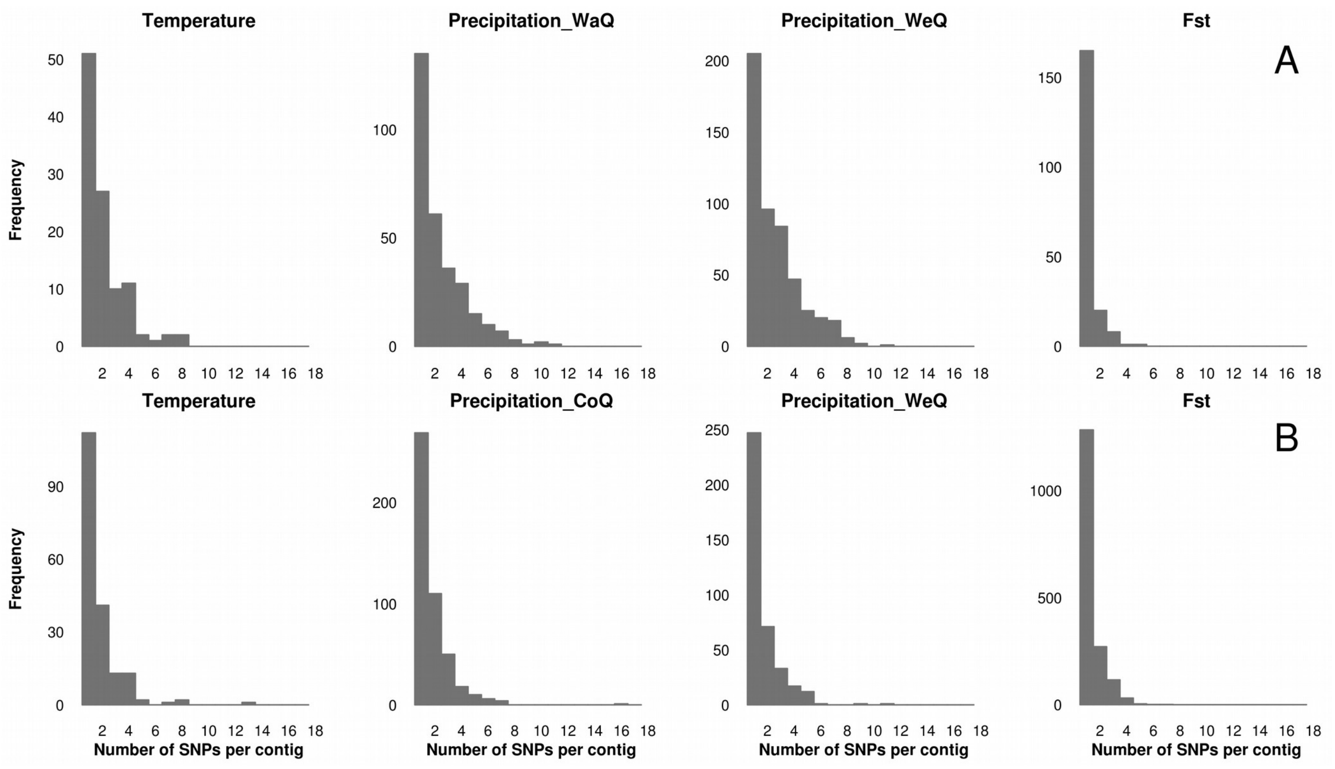

Figure S6: Frequency distribution of the number of SNPs per contig (RAD tag) across independent (non-overlapping) detections in *I. cavalcantei* (A) and *I. maurandioides* (B). Each histogram represents candidate loci identified using environmental association (three environmental variables) and F_{ST} outlier tests.
