# Supplementary material for: Landscape Genomic Conservation Assessment of a Narrow-Endemic and a Widespread Morning Glory From Amazonian Savannas
Source: Front Plant Sci. 2018 May 7;9:532. doi: 10.3389/fpls.2018.00532 (PMC5949356; doi:10.3389/fpls.2018.00532)
Supplement: Supplementary file 15 [file Data_Sheet_1.PDF]

Appendix I

Translated fragments of the *Red Book of the Brazilian Flora* - Martinelli G, Moraes MA (Eds.) (2013) *Livro vermelho da flora do Brasil*. CNCFlora, Centro Nacional de Conservação da Flora, Rio de Janeiro. Available at: http://dspace.jbrj.gov.br/jspui/bitstream/doc/26/1/LivroVermelho.pdf

***Ipomoea cavalcantei* D.F. Austin**

Risk of extinction: Endangered (EN) B1ab (iii,iv).
Evaluator: Solange de Vasconcellos Albuquerque Pessoa.
Date: 17-05-2012.
Distribution: Pará State.
Biome: Amazon.
Justification: Species with a restricted distribution is endemic of the Serra dos Carajás - Serra Norte region, whose native vegetation has been systematically destroyed by the extraction of iron ore. Although the species occurs within a conservation unit, the Carajás National Forest, where it is cited as a common species, this alone does not guarantee the maintenance of its subpopulations. Therefore, there is a clear decline in EOO (Extent of Occurrence) [1], AOO (Area of Occupancy) [2], habitat quality and number of subpopulations.

***Ipomoea carajasensis* D.F. Austin** [3]

Risk of extinction: Vulnerable (VU) D2.
Evaluator: Danielli Cristina Kutschenko.
Date: 17-05-2012.
Distribution: Maranhão and Pará States.
Biomes: Amazon; Cerrado.
Justification: Species with a restricted distribution has an AOO (Area of Occupancy) [2] of 12 km^2 and only three collection records. The specie was registered in two regions of the Campos Rupestres of the Serra dos Carajás (Pará State), whose native vegetation is being irreversibly destroyed mining. The third record was made in the 1980s, near Tocantinópolis (Tocantins State) where little of the original vegetation remains. Therefore, this set of factors represent an immediate risk to the future of this specie's subpopulations.

---

[1] Extent of Occurrence: Area contained within the shortest continuous imaginary boundary which can be drawn to encompass all the known, inferred or projected sites of present occurrence of a taxon, excluding cases of vagrancy (IUCN 2012).
[2] Area of Occupancy: Area within its 'extent of occurrence' which is occupied by a taxon, excluding cases of vagrancy (IUCN 2012).
[3] A recent revision of the genus *Ipomoea* from South America placed *Ipomoea carajasensis* as a synonym of *I. maurandioides*, a widespread species extending from Argentina, Paraguay and Bolivia throughout Brazil (Wood & Scotland 2017). This synonymization implies that this is now a species of *least concern*, as widespread and abundant taxa are included in this category (IUCN 2012). The updated distribution maps of both species are shown below (Fig. 1).

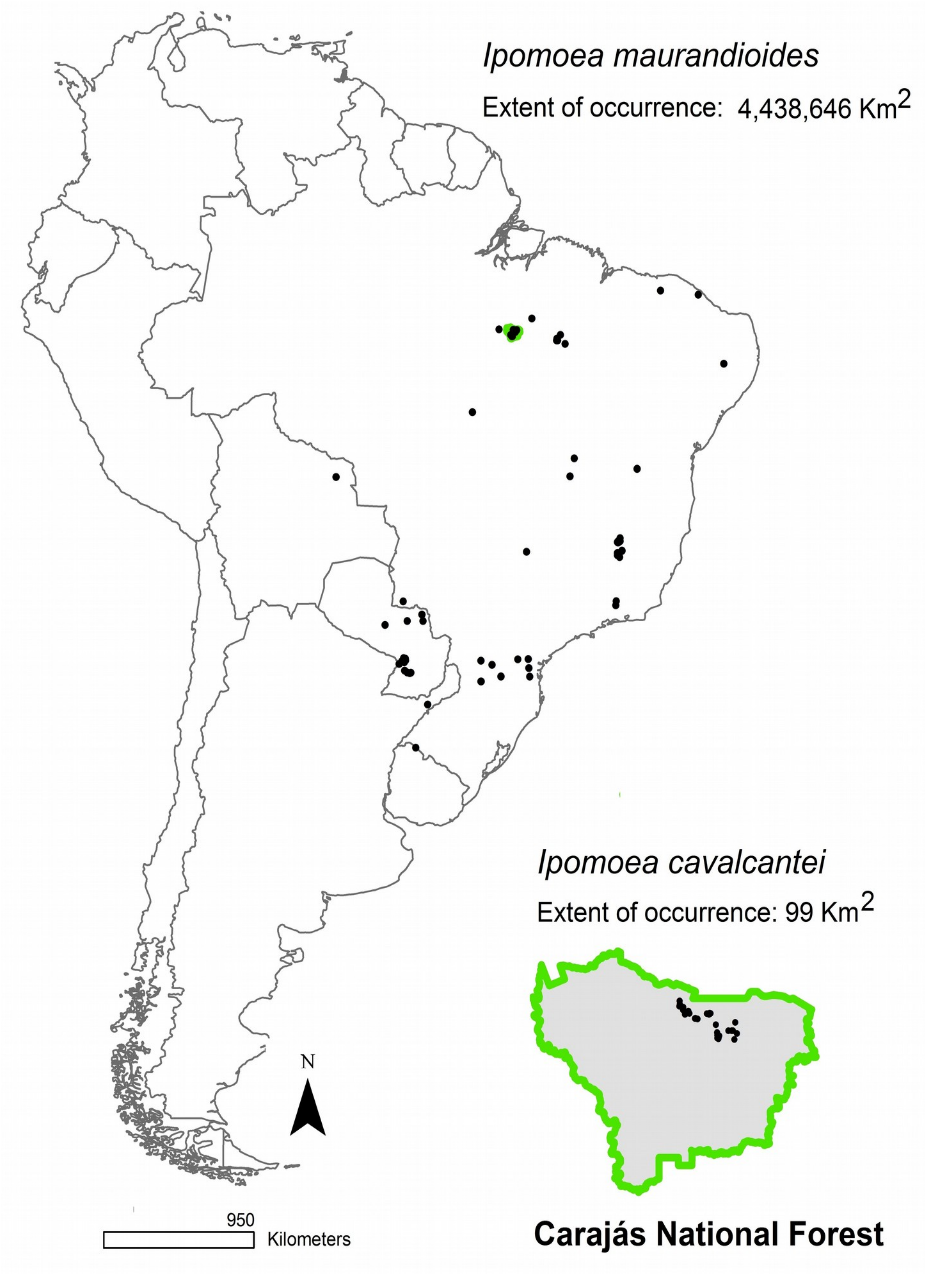

Figure 1: Occurrence records for *Ipomoea maurandioides* and *I. cavalcantei*. Records were retrieved from the Global Biodiversity Information Facility (GIBF: https://www.gbif.org/) and Extent of occurrence was calculated following IUCN specifications (based on the minimum convex polygon containing all the sites of occurrence).
