# Supplementary material for: Landscape Genomic Conservation Assessment of a Narrow-Endemic and a Widespread Morning Glory From Amazonian Savannas
Source: Front Plant Sci. 2018 May 7;9:532. doi: 10.3389/fpls.2018.00532 (PMC5949356; doi:10.3389/fpls.2018.00532)
Supplement: Supplementary file 17 [file Data_Sheet_3.PDF]

Appendix II

Complementary analyses for a subset of *I. maurandioides* samples from Serra Norte (where the species is sympatric with *I. cavalcantei*).

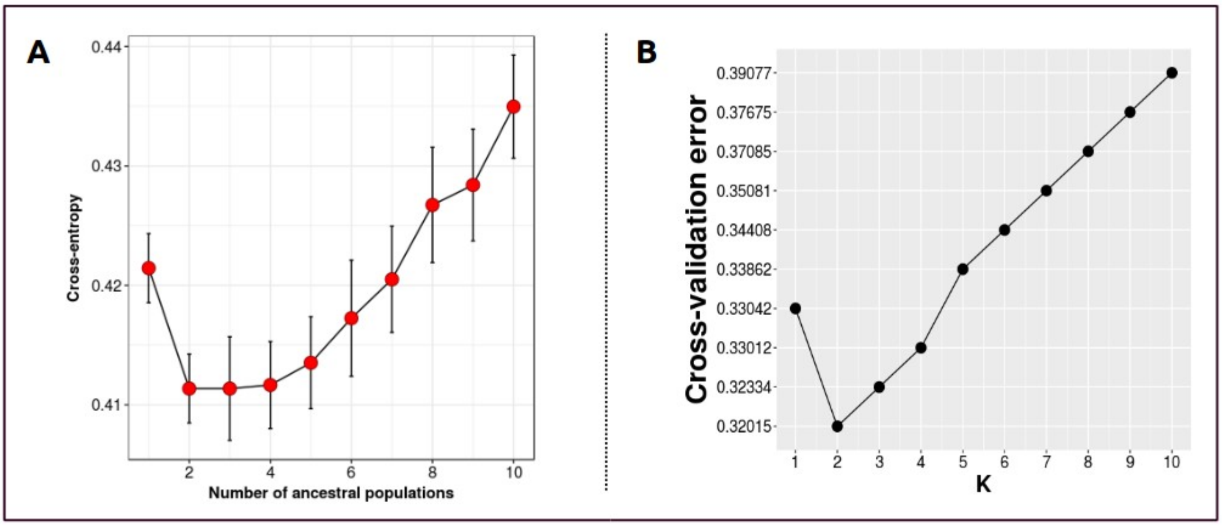

Figure 1: Plots showing the optimal number of genetic clusters (k) for a subset of *I. maurandioides* samples (Serra Norte). Optimal k choice is based on mean ± sd cross-entropy (LEA, A) and cross-validation errors (Admixture, B).

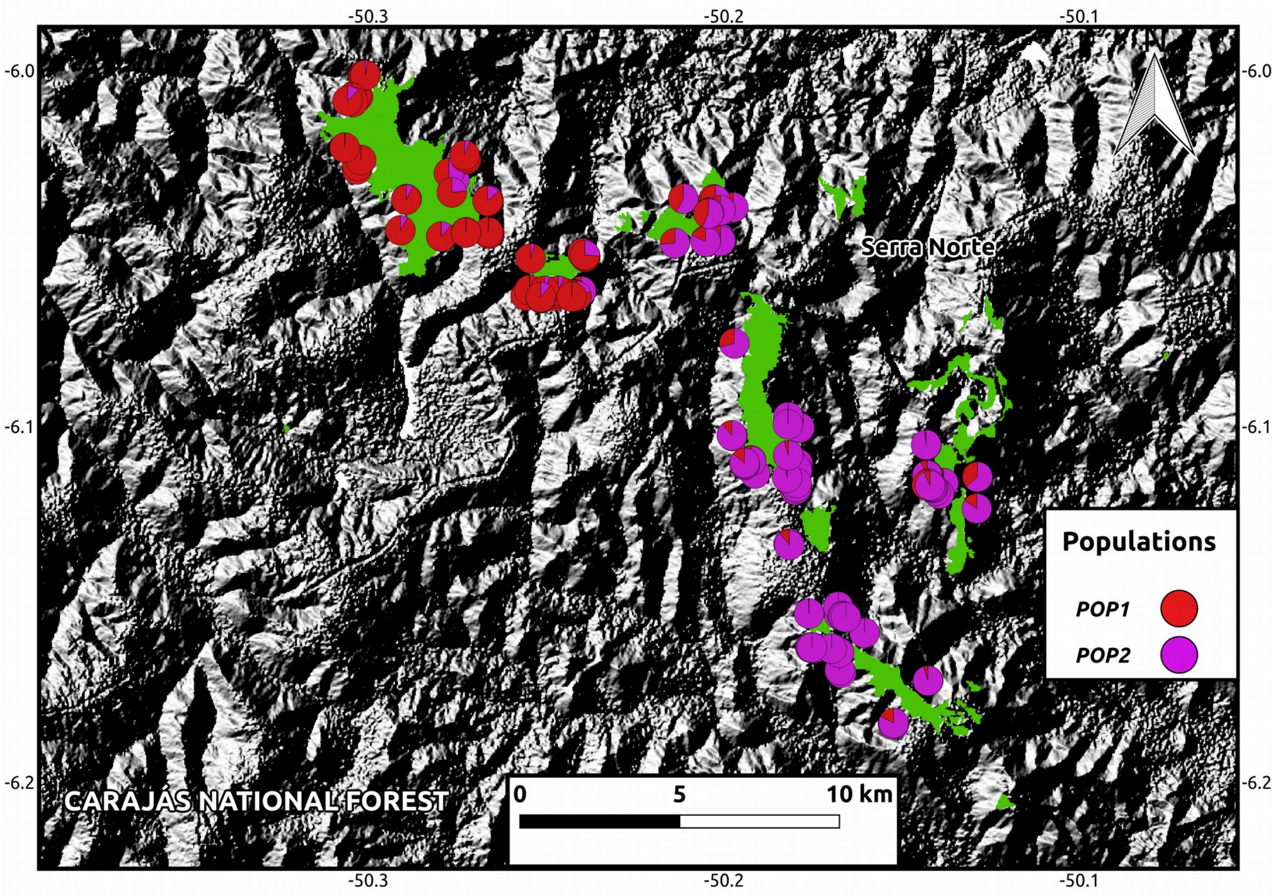

Figure 2: Map showing *I. maurandioides* assignments to two genetic clusters against an elevation (hill shade) layer as background. Pie charts represent ancestry coefficients determined using the LEA package and montane savanna areas are shown in green. The figure shows a subset of *I. maurandioides* samples (Serra Norte).
